# Development landscape of ketohexokinase-C (KHK-C) inhibitors for fructose-driven metabolic disorders: from hit discovery to clinical trials

**DOI:** 10.1039/d6md00645k

**Published:** 2026-10-12

**Authors:** Sri Venkata Gopal Ainapurapu, Timir Naskar, Ajwin Joseph Martin, Bhakti Umesh Hirlekar

**Affiliations:** a Department of Pharmaceutical Chemistry, Manipal College of Pharmaceutical Sciences, Manipal Academy of Higher Education Manipal India bhakti.hirlekar@manipal.edu; b Department of Chemistry and Biochemistry, Thapar Institute of Engineering and Technology Patiala Punjab India

## Abstract

Ketohexokinase (KHK) aka fructokinase, initiates fructose metabolism by catalyzing the ATP-dependent phosphorylation of fructose. Unlike glucose metabolism, which is stringently regulated by numerous hormonal and allosteric mechanisms, fructose metabolism largely bypasses these checkpoints. This is primarily because KHK, unlike glucokinase, is not subject to significant allosteric regulation, resulting in rapid and uncontrolled fructose metabolism. This rapid flux of fructose through the KHK-mediated fructolysis pathway drives the pathogenesis of metabolic dysfunction-associated liver diseases (NAFLD and NASH), obesity, and type 2 diabetes. Moreover, the global consumption of fructose-rich sweeteners and processed foods has increased substantially over the past several decades, amplifying the burden of fructose-driven metabolic disorders (FDMDs). Consequently, KHK has emerged as a promising therapeutic target for the treatment of FDMDs. Genetic deletion and knockdown studies have demonstrated that the suppression of KHK activity protects against FDMDs. Furthermore, pharmacological inhibition of KHK has shown promising efficacy in preclinical and clinical studies, with several candidates demonstrating improvements in the metabolic profile of patients with FDMDs. This review provides an overview of the biological function of KHK in fructose metabolism and its involvement in metabolic diseases. It also discusses the structure and catalytic mechanism of KHK and provides a detailed overview of KHK inhibitor discovery and development, from initial hit identification and lead optimization to preclinical development and clinical evaluation.

## Abstract 2

Excessive dietary fructose intake promotes ketohexokinase (KHK)-mediated fructose-driven metabolic disorders (FDMDs). Targeting KHK with small-molecule inhibitors is emerging as a potential therapeutic strategy for the treatment of FDMDs.

## Graphical abstract

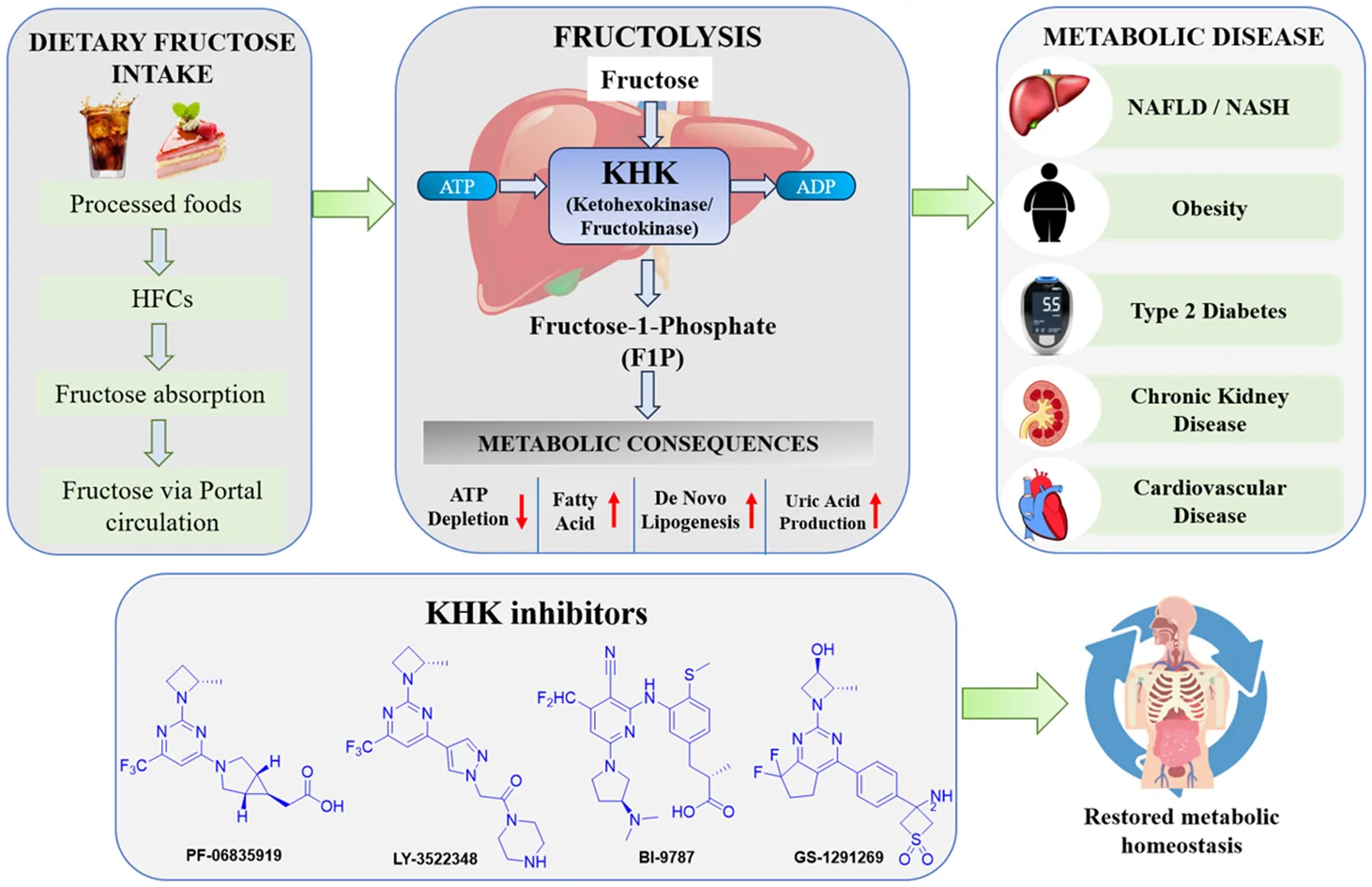

## 1. Introduction

Dietary patterns in both industrialized and developing countries have changed substantially over the past two decades.^1,2^ Although safe daily fructose intake is estimated to be approximately 25–40 g, the widespread consumption of ultra-processed foods and sugar-sweetened beverages has increased average fructose intake to 85–100 g per day or even higher in some populations.^3–7^ A major contributor to this increase is the extensive use of high-fructose corn syrup (HFCS) as a sweetener in ultra-processed foods.^8–10^ High intake of fructose has been identified as a significant factor in metabolic disorders including non-alcoholic fatty liver disease (NAFLD), non-alcoholic steatohepatitis (NASH), obesity, type 2 diabetes mellitus (T2DM), and cardiovascular disease.^7,11–18^

Ketohexokinase (KHK) aka fructokinase, is the primary and rate-limiting enzyme in fructose metabolism.^19^ In the liver, KHK initiates fructose metabolism by phosphorylating it to fructose-1-phosphate (F1P). F1P consequently enters multiple metabolic pathways involved in energy production, lipogenesis, and nucleotide synthesis. Unlike glucose metabolism, fructose metabolism is largely devoid of regulatory control at its initial conversion step.^20,21^ As KHK is not subject to allosteric regulation, high fructose concentrations rapidly increase metabolic flux through downstream glycolytic and lipogenic pathways. This unregulated fructose metabolism promotes the rapid conversion of fructose into triglycerides, leading to hepatic lipid accumulation playing a key role in the pathogenesis of NAFLD^18^ and NASH.^22^ Furthermore, fructose metabolism stimulates *de novo* purine synthesis and ATP depletion, resulting in increased production of uric acid.^13,23,24^ Elevated uric acid levels can lead to systemic hyperuricemia, which is associated with hypertension, insulin resistance, visceral adiposity, and chronic kidney disease.^25^ Collectively, these metabolic disturbances contribute to the development of cardiometabolic disorders, including T2DM and cardiovascular disease.^12,15,26^ These observations highlight the central role of KHK in fructose-driven metabolic disorders (FDMDs) and support its potential as a therapeutic target.

The role of KHK in metabolic diseases is further supported by several studies that have demonstrated the therapeutic benefits of KHK inhibition. Genetic deficiency of both KHK isoforms completely protected mice against FDMDs.^27^ Selective knockdown of the hepatic KHK-C isoform has recently been shown to protect against fructose-induced obesity, fructose-induced steatosis, and NAFLD.^28–30^ Together, these findings provide strong evidence that KHK, particularly the KHK-C isoform, plays a critical role in FDMDs and represents a promising therapeutic target for the treatment of metabolic disorders. To date, nearly two decades of research have led to the development of four KHK inhibitors that have progressed to preclinical or clinical evaluations for the treatment of FDMDs. This includes PF-06835919^31^ and LY-3522348,^32^ which have completed phase 1 clinical trials, while BI-9787^33^ and GS-1291269^34^ remain in preclinical development for the treatment of NAFLD/NASH, type 2 diabetes, obesity, and hereditary fructose intolerance. KHK inhibitors are emerging as potential therapeutic agents for the treatment of fructose-mediated metabolic disorders, particularly NAFLD and NASH (discussed in the KHK inhibitor section). Although the biological role of KHK has been extensively studied, medicinal chemistry and drug discovery efforts targeting KHK have not yet been comprehensively reviewed.

From the perspective of KHK inhibitor discovery, this review highlights the role of KHK in metabolic diseases, its structure and catalytic mechanism, and the discovery and development of KHK inhibitors from early leads to clinical candidates. In addition, translational gap, selectivity issue, and future directions in the development of KHK inhibitors are summarized in this review.

## 2. Role of KHK in fructose-driven metabolic disorders (FDMDs)

Glycolysis occurs throughout the body and is highly regulated by phosphofructokinase-1 (PFK-1), which is insulin-dependent.^19,35^ In contrast, fructose metabolism begins with phosphorylation by the hepatic KHK-C enzyme, bypassing PFK-1, which is insulin-independent.^14^ In hepatocytes, KHK-C initiates fructose metabolism by phosphorylating fructose to fructose-1-phosphate (F1P), which is subsequently metabolized by aldolase B into dihydroxyacetone phosphate (DHAP) and glyceraldehyde (see Fig. 1).^36^ Both metabolites are converted to glyceraldehyde-3-phosphate, which enters glycolysis and is metabolized to pyruvate.^14,37^ Pyruvate is further converted to acetyl-CoA, a key substrate for *de novo* fatty acid synthesis in the tricarboxylic acid (TCA) cycle.^38^ In parallel, glyceraldehyde and DHAP act as direct precursors for glycerol-3-phosphate synthesis, providing the glycerol backbone for triglyceride formation.^17,39^ As a result, largely unregulated fructose metabolism rapidly converts fructose into triglyceride independent of cellular energy status. Together, this increased fatty acid production and triglyceride assembly drive hepatic lipid accumulation, promoting inflammation and contributing to the development of NAFLD.^18^ This excess hepatic fat is a prerequisite for NASH and predisposing the liver to further inflammation and injury.^22^

**Fig. 1:**
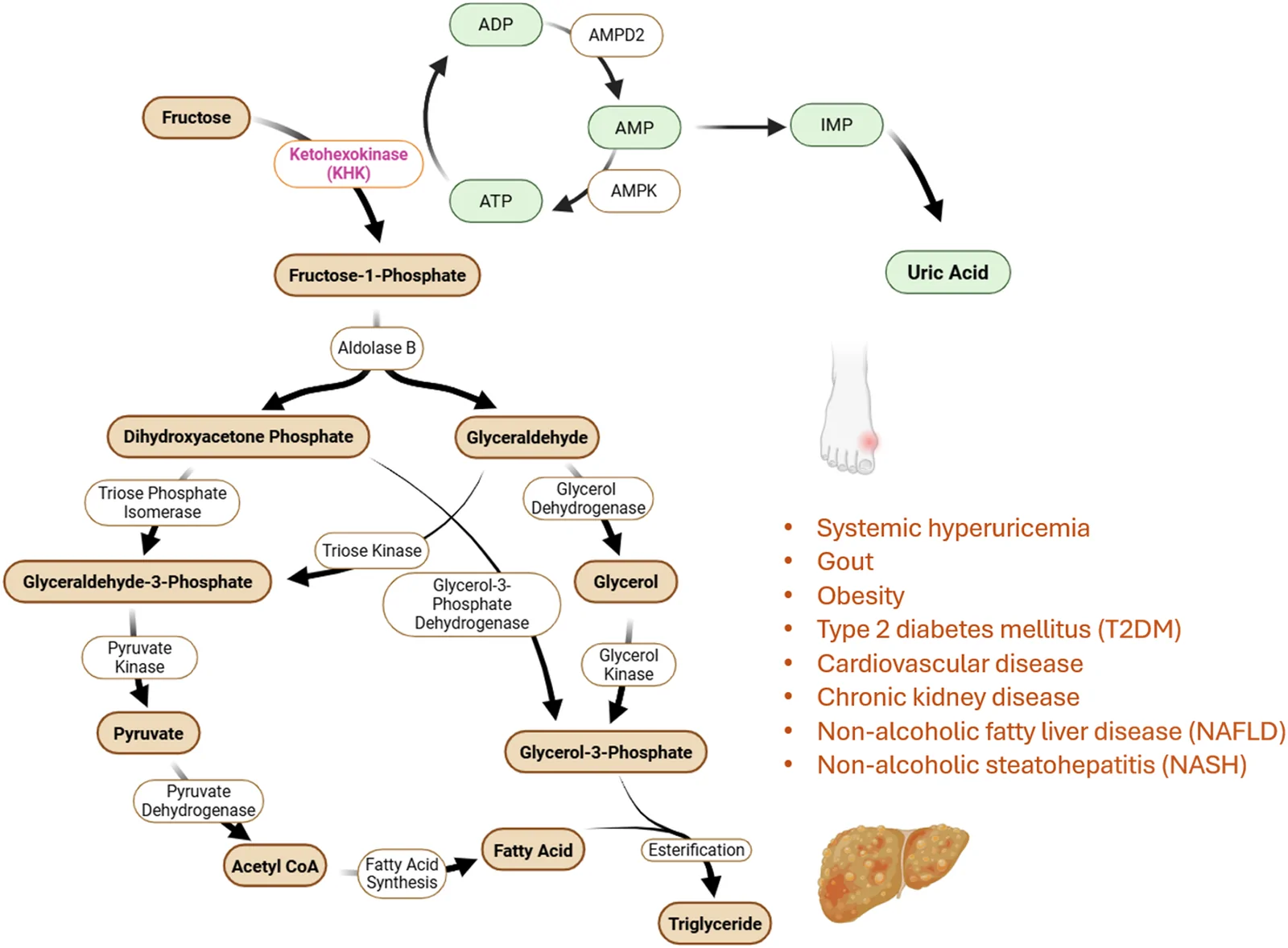
Overview of the fructolysis pathway and metabolic end products associated with metabolic disorders.

Concurrently, fructose metabolism *via* KHK-C induces rapid, concentration-dependent depletion of intracellular ATP and inorganic phosphate.^40^ Inorganic phosphate normally inhibits AMP deaminase (AMPD2), the rate-limiting enzyme in purine degradation and uric acid formation.^14^ Thus, phosphate depletion removes this inhibitory constraint, activating AMPD2 and accelerating uric acid production. Fructose also promotes *de novo* purine synthesis, further increasing uric acid levels.^13,23,24^ This excess uric acid gets crystallize within synovial joints, causing gout, while systemic hyperuricemia contributes to secondary risk factors such as hypertension, diabetes, and visceral adiposity and is strongly associated with a range of cardiometabolic disorders.^25^

In contrast to well-established glucose metabolism, KHK does not undergo feedback inhibition or allosteric regulation. Due to the largely unregulated nature of fructose metabolism, it does not induce insulin secretion from pancreatic β-cells. This leads to a decrease in circulating insulin and leptin, while rise in ghrelin levels.^20,21^ These hormones play central roles in the long-term regulation of appetite and energy balance through signaling to the central nervous system.^41,42^ On the other hand, glucose metabolism elevates the ATP/ADP ratio, closes KATP channels, induces membrane depolarization, and triggers calcium-dependent exocytosis of insulin granules.^43^ In contrast, fructose metabolism does not generate sufficient ATP to initiate this signaling cascade, and insulin granule exocytosis does not occur.^15^ Consequently, the fructose-induced dysregulation of insulin, leptin, and ghrelin, together with ATP depletion, reduces satiety and increases hunger.^41,44,45^ Over time, chronic intake of high-fructose diets increases overall caloric consumption and promotes weight gain and obesity.^46,47^ Thus, lack of regulatory control in fructose metabolism contributes to NAFLD/NASH, cardiovascular diseases due to the combined effect of hepatic lipid accumulation, uric acid-mediated mitochondrial stress, and obesity-associated hormonal dysregulation, impaired insulin signalling, and susceptibility to type 2 diabetes.^12,15,26^

## 3. KHK isoforms: structural similarities and differences

Fructokinase or ketohexokinase (KHK; UniProt: P50053)^48^ belongs to the phosphofructokinase B (PfkB) family, also known as the carbohydrate kinase (ribokinase) family.^49^ It consists of a 298-amino-acid monomer and is structurally distinct from classical hexokinases.^49^ It exists as two splice isoforms (KHK-A and KHK-C), which differ in exon 3, resulting in a 44-amino-acid sequence variation corresponding to residues 72–115 (see Fig. 2A).^50,51^ Sequence alignment showed 90.60% similarity between the two isoforms, with a highly conserved catalytic site. However, structural difference caused by the alternatively spliced region affects the relative orientation of the β-clasp and central α/β domain.^50,51^ KHK-A is ubiquitously expressed and exhibits relatively low affinity for fructose (*K*_m_ ≈ 8 mM), whereas KHK-C, predominantly expressed in metabolic tissues such as the liver, kidney, and intestine, displays a markedly higher affinity for fructose (*K*_m_ ≈ 0.8 mM).^52^ This difference in substrate affinity makes KHK-C therapeutically more relevant despite the highly conserved catalytic architecture of the two isoforms (hereon discussion is mainly focused on KHK-C isoform).

**Fig. 2:**
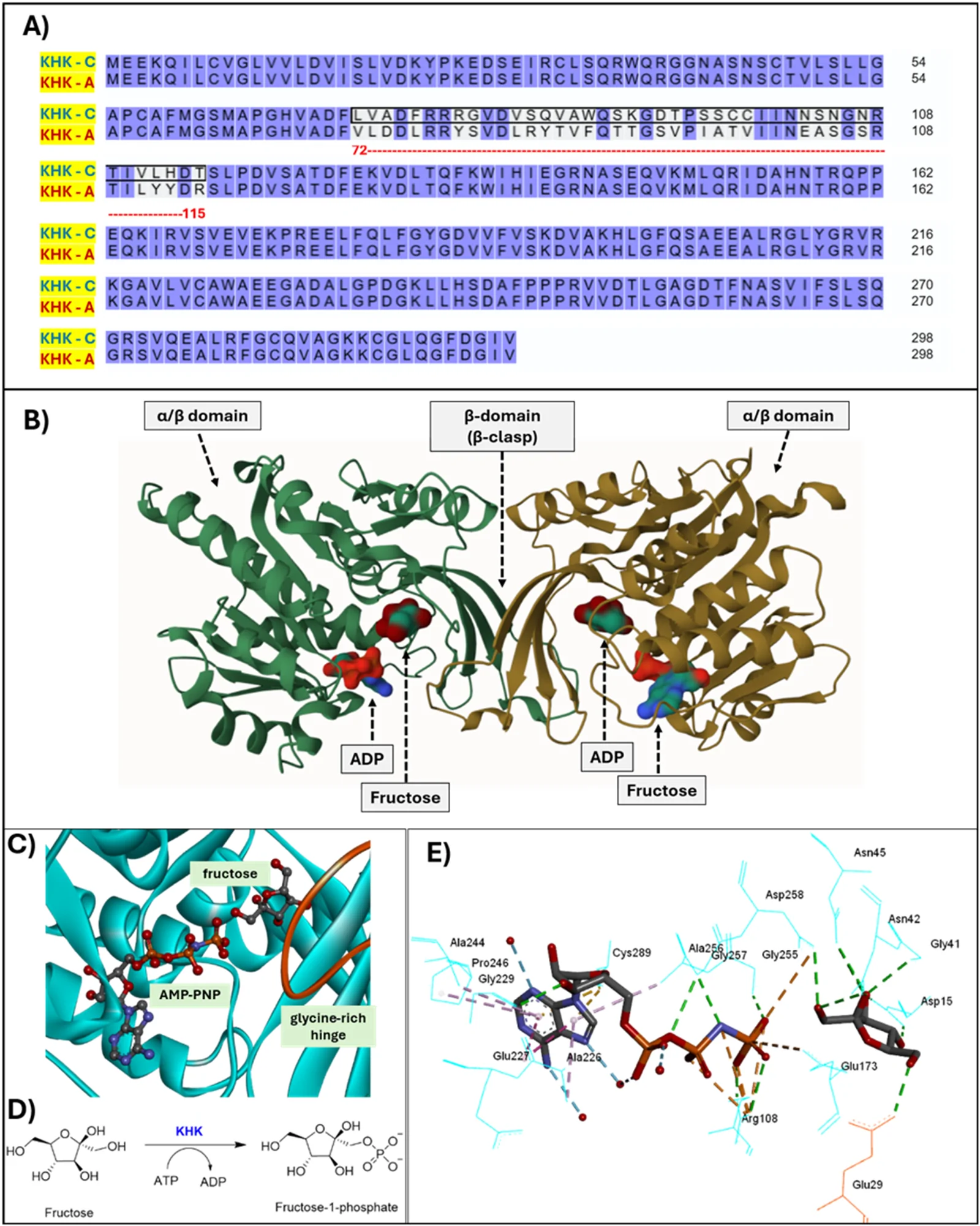
(A) Sequence alignment of the KHK-C and KHK-A isoforms showing the alternatively spliced region. (B) Structure of the ketohexokinase (KHK) homodimer bound to ADP and fructose (PDB ID: 6P2D). Structural domain: β clasp an α/β domain. (C) PDB ID: 3BNV; catalytic domain of KHK showing AMP-PNP and fructose bound within the active site. Also, showing the glycine-rich loop (residues 25–31) of monomer B protrudes into the active site of monomer A. (D) Reaction showing KHK-catalyzed phosphorylation of fructose to fructose-1-phosphate (F1P). (E) 3D view of AMP-PNP and fructose interactions with key amino acid residues at the KHK active site (PDB ID: 3BNV).

## 4. Structure and catalytic mechanism of KHK-C

The biologically relevant form of KHK is a homodimer stabilized by two pairs of identical intermolecular interactions.^53^ The tertiary structure of each monomer unit of KHK-C is organized into two domains: a large central α/β domain and a β-domain (β-clasp) (see Fig. 2A). The central α/β domain contains a Rossmann-like fold consisting of a nine-stranded antiparallel β-sheet flanked on both sides by five α-helices.^54^ The β-sheet domain is composed of a four-stranded β-sheet and acts as a lid over the active site of the neighbouring monomer (see Fig. 2B). The protruding β-sheets of the lid domains from each monomer interact orthogonally, generating an overall *C*_2_ symmetry. At the dimer interface, a β-strand from one monomer pairs with the corresponding β-strand from the other through intermolecular hydrogen bonding, forming a β-clasp that stabilizes the dimeric assembly.^51,53^ These two domains are connected by an N-terminal glycine-rich hinge containing two consecutive glycine residues, enabling the movement of the lid relative to the central domain (see Fig. 2C). The ATP-binding and catalytic sites are positioned within the cleft between the α/β core and lid domains. The lid closes over this cleft upon substrate binding to stabilize the catalytic conformation of the enzyme.

In the presence of ATP and magnesium, KHK facilitates the metabolism of fructose by adding a phosphate group to d-fructose at the C1 position, resulting in the formation of fructose-1-phosphate (F1P) (see Fig. 2D).^36,53^ As previously mentioned, KHK has a dimeric conformation with monomer A being the active component for catalysis. Within the ATP-binding region of the active site of monomer A: Asp258 and the β-clasp residue Arg108 are key residues necessary for the catalytic process (see Fig. 2E).^50,51^ The Asp258 acts as a conjugate base and abstracts a proton from the 1-OH group of d-fructose, forming a nucleophilic alkoxide ion (1-O^−^).^55^ This formed alkoxide ion (nucleophile) attacks the γ-phosphate of ATP, which is positioned by Arg108 within the active site.^53^ Additionally, Asp27B from glycine-rich loop of monomer B extends into the active site of monomer A, positioning itself about 6 Å from the catalytic center.^52^ Although its role in catalysis is unclear, it may contribute to the stabilization of the active site.

## 5. Therapeutic significance of KHK-C

The central role of KHK in fructose metabolism and its contribution to the development of metabolic disorders make it an attractive therapeutic target. This is validated by a recent study showing that KHK expression was increased in fructose-fed mice and obese patients with steatohepatitis.^29^ Furthermore, numerous studies have demonstrated that KHK inhibition can prevent or attenuate the development of FDMDs. This was supported by selective knockdown of hepatic KHK-C in mice which protected against fructose-induced steatosis.^28,29^ However, genetic deletion of both KHK-A/C isoforms conferred complete protection against FDMDs, including obesity and insulin resistance.^27^ Conversely, researchers also found that deletion of KHK-A alone exacerbated fructose-induced metabolic abnormalities in mice.^56^ It is evident that KHK-A facilitates excess fructose clearance and limits its availability for metabolism by KHK-C. Hence, it may modulate fructose metabolism and have protective role in FDMDs.^27^ However, another study reported that selective inhibition of KHK-C, but not KHK-A, protected animals from FDMDs.^57^ Although these genetic studies suggest that KHK-C is the major therapeutic target; KHK-A and KHK-C deletion showed contrasting effects. It raises an important question concerning the therapeutic significance of isoform selectivity. Nonetheless, these collective findings have established KHK-C as an attractive therapeutic target for drug discovery. Consequently, several small-molecule KHK inhibitors have been developed, with a few advancing to clinical trials for the treatment of metabolic diseases.

## 6. Discovery of KHK inhibitors

This section provides an overview of the discovery and development of preclinical and clinical KHK inhibitors. It discusses the progression from initial hit identification through lead optimization to clinical candidate selection using medicinal chemistry strategies.

### Early lead identification of KHK inhibitors

A fragment-based drug design (FBDD) approach guided by electron density mapping was employed by Johnson & Johnson Pharmaceutical Research and Development.^58,59^ X-ray diffraction (XRD)-guided analysis of fragment electron density within the ATP-binding site of KHK was performed. This led to the discovery of lead-like inhibitors without using affinity data. Primary screening led to the identification of pyrazole-4-carboxamide fragments (1 and 2, see Fig. 3). Further fragment optimization revealed that modifications to the *S*-methyl and *N*-phenyl substituents were not tolerated. XRD analysis revealed that Asp-27B (monomer B) is a catalytically significant residue within the fructose-binding site. By strategically expanding the pyrazole core toward this region, fragment-to-hit optimization cycles led to the identification of an indazole core scaffold (3; IC_50_ < 5 μM). Subsequent hit-to-lead optimization was driven by considerations based on molecular size, binding orientation, shape complementarity, synthetic tractability and patentability.

**Fig. 3:**
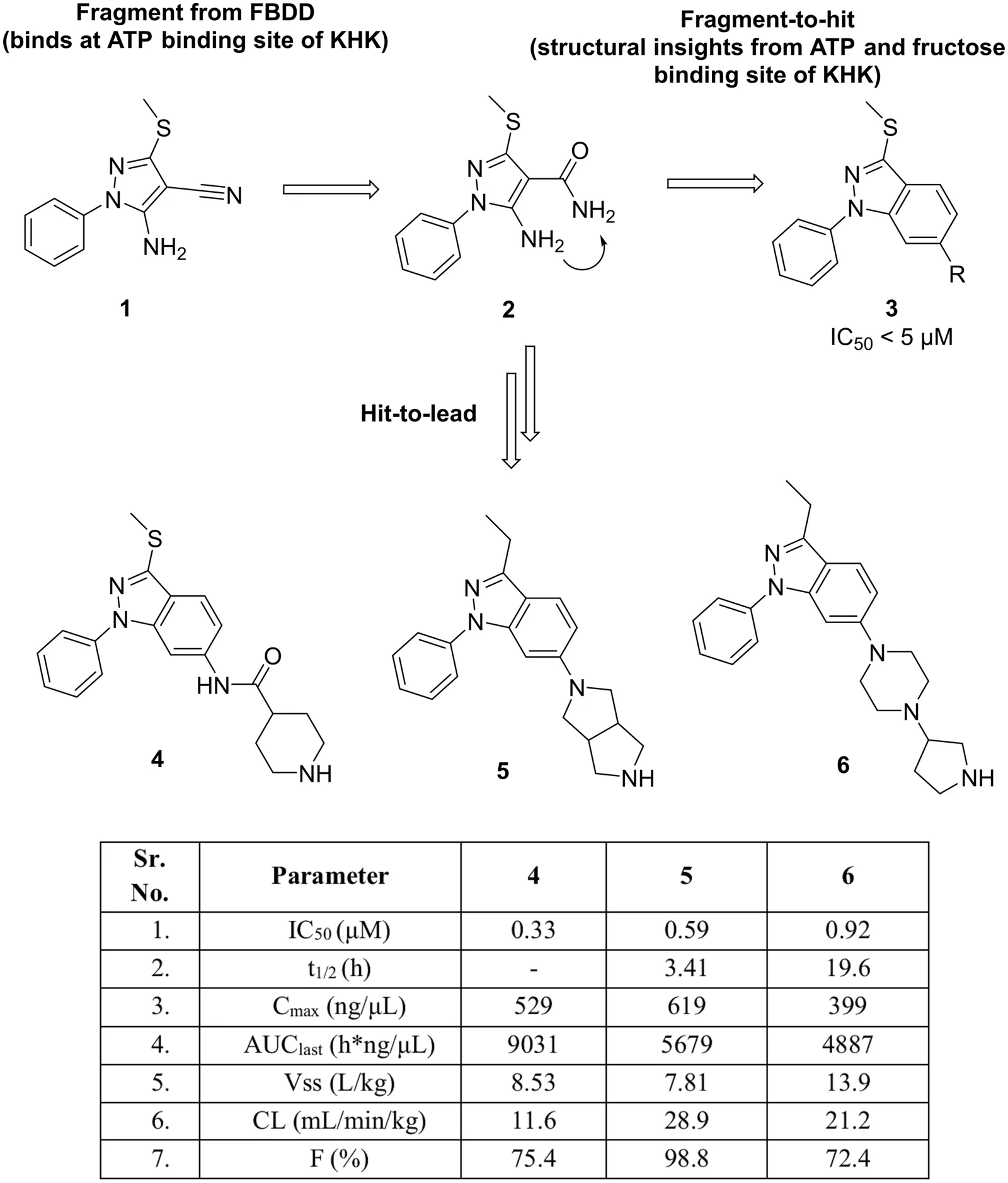
Development of lead candidates 4, 5, and 6*via* fragment-based drug discovery.

Compounds 4, 5, and 6 were selected as lead candidates as they exhibited sub-micromolar potency in a KHK enzyme assay (IC_50_ = 0.33, 0.59, and 0.92 μM, respectively), showed no significant inhibition of major CYP enzymes, and demonstrated high metabolic stability in both human and rat liver microsomes. Similarly, *in vivo* studies have demonstrated high oral bioavailability in rats (*F* > 70% at 10 mg kg^−1^), along with moderate systemic clearance and a high volume of distribution. Safety and selectivity assessments revealed moderate hERG inhibition and minimal off-target activity, with notable on-target inhibition of the KHK-A isoform. In addition, compound 4 displayed favorable physicochemical properties (high aqueous solubility) and good permeability in Caco-2 cells. Overall, all these compounds exhibited favorable pharmacokinetic and selectivity profiles in rats.

In 2011, the same research group identified the pyrimidino[5,4-*d*]pyrimidine chemotype as a promising starting point through a high-throughput screening (HTS) campaign of approximately 800 000 structurally diverse compounds.^60^ Subsequently SBDD of the pyrimidino[5,4-*d*]pyrimidine core led to the identification of compound 7 (see Fig. 4) with submicromolar potency (IC_50_ = 400 nM). The optimal activity of compound 7 was observed with a piperazine substituent at *N*^2^, a 2-methylphenyl group at *N*^4^, and a methyl group at *N*^8^ of the pyrimidino[5,4-*d*]pyrimidine core. Further optimization at the R_1_ position revealed that replacing the *N*^8^ methyl group with an NH-cyclopropylmethyl substituent significantly improved potency (compound 8; IC_50_ = 210 nM). However, R_2_*ortho*-modification from an *N*^4^-2-methyl substituted phenyl group (compound 8) to an unsubstituted phenyl group resulted in a substantial loss of activity (compound 9, IC_50_ = 3200 nM). In contrast, moderate substitution with a *N*^4^-2-methoxyphenyl group (compound 10; IC_50_ = 100 nM) and a *N*^4^-2-methylthiophenyl group (compound 11, IC_50_ = 12 nM) showed significantly enhanced potency. Owing to its potent intrinsic and cellular inhibitory activities, compound 11 was advanced for further pharmacological evaluation, including off-target assessment and ADME studies. Lead compound 11 exhibited greater than 50-fold selectivity over three off-target metabolic kinases (ribokinase, adenosine kinase, and hexokinase) without significantly inhibiting major CYP enzymes. Furthermore, compound 11 exhibited good physicochemical properties, such as high aqueous solubility and permeability in Caco-2 cell experiments. *In vivo* studies have shown moderate oral bioavailability in rats (34% at 10 mg kg^−1^), along with high systemic clearance (CL = 160 mL min^−1^ kg^−1^) and large volume distribution (Vd_ss_ = 32 L kg^−1^).

**Fig. 4:**
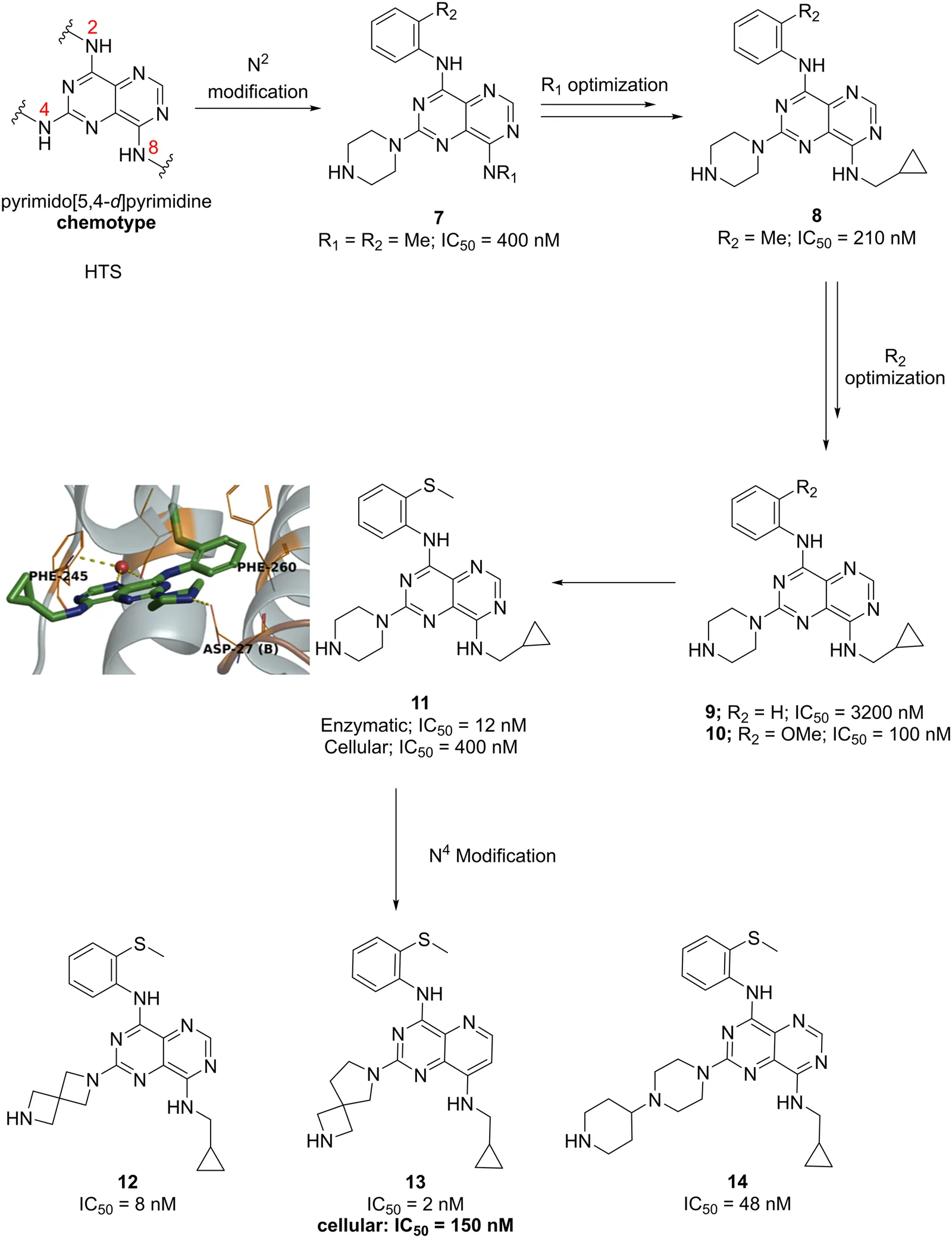
Development of pyrimidino[5,4-*d*] pyrimidine-based compounds 12, 13, and 14.

Additionally, the reported crystal structure of compound 11 in complex with KHK revealed hydrogen-bonding interactions between *N*^3^ and the conserved water molecule, while the *N*^2^ phenyl-*S*-methyl group showed hydrophobic interactions with Phe-260A at the hydrophobic cleft. Moreover, the *N*^4^ protonated piperazine moiety forms a salt bridge with the carboxylate side chain of Asp-27B (see Fig. 4; binding pose of compound 11).

Given the catalytic role of Asp27B in anchoring the ligand within the active site. Compound 11 was modified at the terminal ammonium group attached at *N*^4^ position to enable extension into the “triphosphate channel” of monomer B to maximize additional favorable interactions.^61^ A series of substituents bearing ammonium functionalities were introduced at the *N*^4^ position of the pyrimidino[5,4-*d*]pyrimidine scaffold, while retaining the *N*^2^ phenyl-*S*-Me and *N*^8^ NH-cyclopropylmethyl substituents of compound 11. The incorporation of a bicyclic piperazine, a fused phenyl ring, or a bulky bicyclic amine, as well as the introduction of conformational constraints, adversely affected potency (IC_50_ > 0.8–6 μM). In contrast, bioisosteric replacement with a spirocyclic piperazine, namely, a 2,6-diazaspiro[3.3]hept-2-yl group (compound 12; IC_50_ = 8 nM) and 2,6-diazaspiro[3.4]octan-6-yl (compound 13; IC_50_ = 2 nM) at the *N*^4^ position. Surprisingly, compound 14 bearing a longer substituent at *N*^4^ that does not extend into the vicinity of Asp27B, also retained substantial activity (IC_50_ = 48 nM).

Compound 14 was further evaluated against a diverse panel of 31 human protein kinases, and none exhibited greater than 50% inhibition at 10 μM concentration. *In vivo* PKPD studies have not been reported for this series. Pyrimidino[5,4-*d*]pyrimidines and indazoles have not been reported to inhibit rodent KHK *in vitro*. However, the high metabolic clearance of these chemotypes resulted in low systemic exposure in rat pharmacokinetic (PK) studies. This may be due to the presence of basic amine functionality, which is key in both series.

To address the issues associated pyrimidino[5,4-*d*]pyrimidine and indazole containing scaffold, NMR-STD guided FBDD method was used to screen the Pfizer proprietary fragment library.^62^ Their screening gave approximately 500 primary fragment hits with reliable binding affinity which were further narrow downed by SPR assay. A total of 23 promising neutral fragments with reliable binding affinities (*K*_D_) were prioritized for XRD analysis. All fragment hits were found to have substituted heterocyclic core which occupies both adenine and ribose binding pockets within the ATP-binding site. In the ribose-binding pocket, most fragments feature a saturated ring bearing a polar substituent at the 12 o'clock position, forming a hydrogen bond with the backbone carbonyl oxygen of Gly229. In the adenine-binding pocket, a nitrile group at the 10 o'clock position interacts with a conserved water molecule, whereas lipophilic substituents at the 8 o'clock position form hydrophobic interactions. The Pfizer library was rescreened to identify compounds bearing aryl cores with substituents capable of satisfying all three key interactions.

This resulted in the identification of compounds 15 and 16, bearing pyrrolidine and piperidine chemotypes, which displayed hKHK IC_50_ values of 0.67 μM and 16.79 μM, respectively (see Fig. 5). Both compounds showed binding within the ATP-binding pocket of the enzyme, maintaining the key interactions required for activity. However, compound 15 exhibited good inhibitory potency and metabolic stability (HLM CL < 8 μL min^−1^ mg^−1^) but low systemic exposure in rats. In contrast, compound 16 was a weaker inhibitor but offered a modifiable structural position; therefore, the authors merged the pharmacophore features. Resulted lipophilic compound 17 with reasonable enzymatic inhibitory potency (IC_50_ = 1.03 μM) with improved metabolic stability in HLM. In subsequent optimization cycles, modification of the piperazine vector of compound 17 with neutral analogues resulted in the identification of a *trans*-dihydroxypyrrolidine derivative with submicromolar potency (18; IC_50_ = 0.45 μM).^63^

**Fig. 5:**
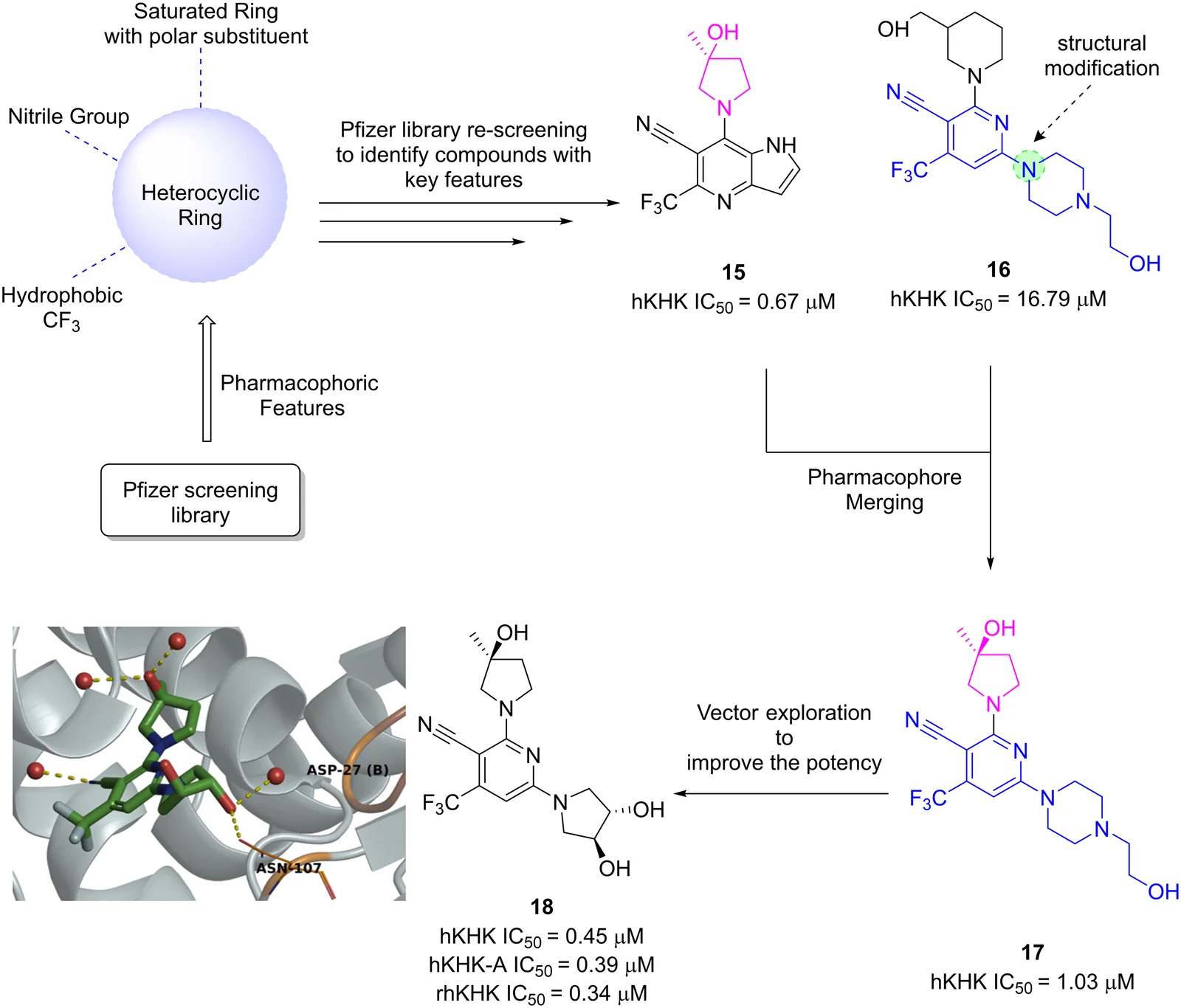
Development of indazole-containing compound 18 by Pfizer.

These efforts led to the identification of a potent and stable compound with an overall improved profile (CL < 8 μL min^−1^ mg^−1^). Additionally, acceptable oral bioavailability (*C*_max_ = 2.94 μg mL^−1^, AUC_last_ = 14.5 μg h mL^−1^) and systemic clearance (25.9 mL min^−1^ kg^−1^) were observed in rats at an oral administration of 5 mg kg^−1^ dose. PK studies in rats demonstrated a moderate volume of distribution (1.38 L kg^−1^) and a short half-life (1.2 h). Furthermore, it showed KHK-C selectivity, with no significant activity against hERG, CYP450 enzymes, and broad panel of transporters. Moreover, no genotoxicity or cytotoxicity was observed under the tested conditions, indicating its suitability for further optimization.

### Lead optimization and preclinical development of KHK inhibitors

Pfizer continued to optimize compound 18 to improve its potency and physicochemical properties. The substitution of the (*S*)-3-methylpyrrolidin group with a (2*S*,3*R*)-2-methylazetidin, located 19 deeper within the ribose pocket, preserves essential hydrophobic interactions and retains comparable potency (see Fig. 6). Subsequent efforts to avoid the basic amine character and minimize the interaction with Asp27B involved the exploration of diverse amines oriented proximally to Arg108 (β-clasp residue). This afforded lead compounds 20 and 21 with ∼20-fold improved potency, where the pyrrolidine-3,4-diol was replaced by a 3-azabicyclo[3.1.0]hexane moiety bearing a terminal carboxylic acid. Although these compounds showed an improved overall profile, including moderate cell permeability and good metabolic stability in HLM, but exhibited high turnover in human hepatocytes. Additionally, metabolite identification studies in humans and rats indicated glucuronidation liability at the secondary hydroxyl group.

**Fig. 6:**
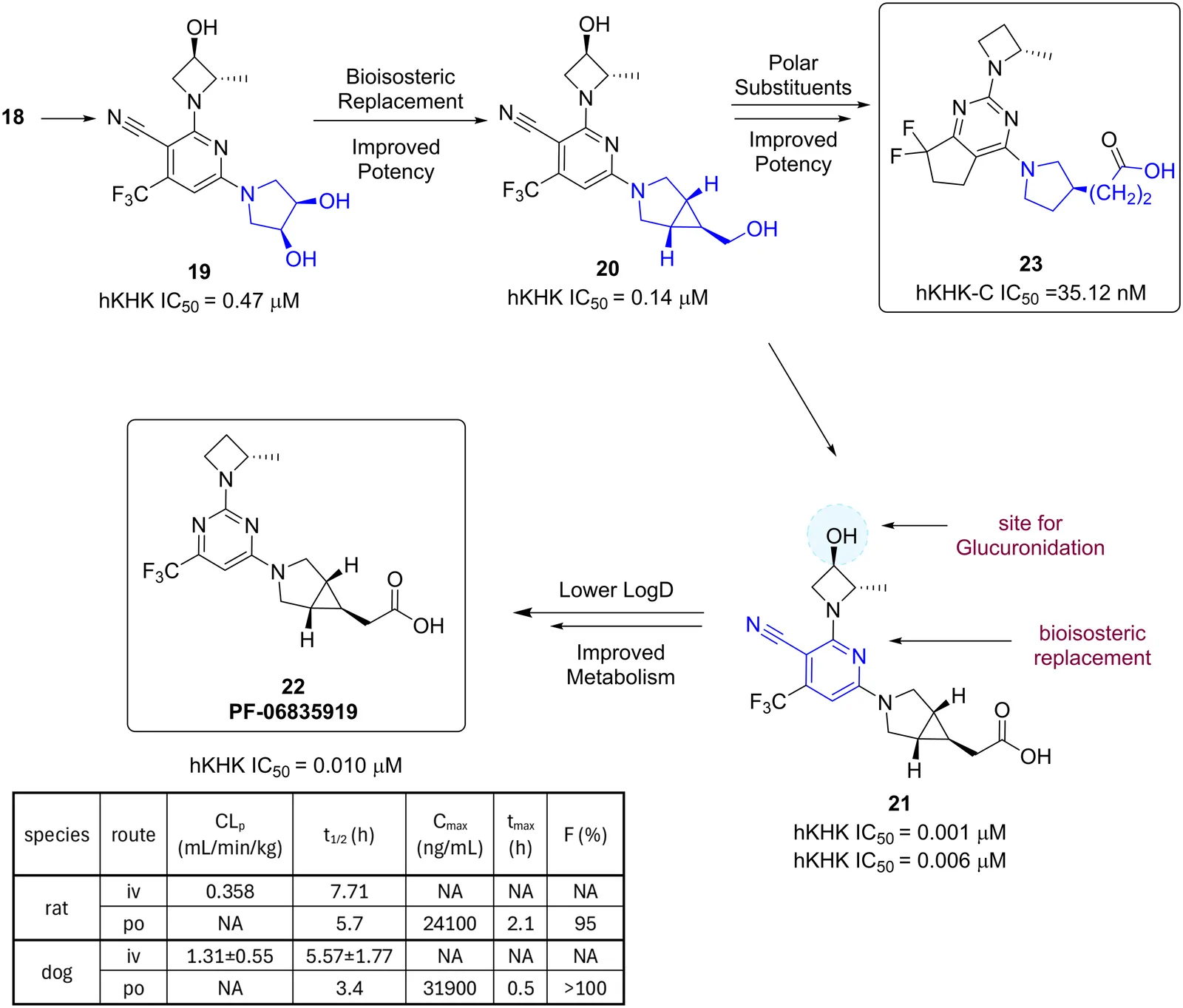
Development of clinical candidate PF-06835919:3-azabicyclo[3.1.0]hexane-substituted pyrimidine-type compound.

To further improve potency and metabolic profile, the cyanopyridine group was replaced with a pyrimidine core, and the hydroxyl group from the azetidine ring was removed. Through these collective efforts, the first-in-class KHK inhibitor PF-06835919 (22) was developed with an improved overall profile (see Fig. 1).^31^ PF-06835919 exhibited an excellent pharmacokinetic profile in both rats and dogs, characterized by low systemic clearance, and high oral exposure (half-life of 7.71 h, *C*_max_ of 24 100 ng mL^−1^, oral bioavailability of 95%). PF-06835919 has completed phase 1 clinical studies for the treatment of NAFLD/NASH; no report on the completion of the phase 2 clinical trial has been published to date.^64^

The TuoJie Biotech group also attempted to improve the KHK inhibitor affinity by modifying polar groups to enhance ionic interactions with Arg108. The compound also exhibited a favorable pharmacokinetic profile in preclinical studies. In rats, compound 23 showed a half-life of 7.16 h, and oral bioavailability of 54.6%.^65^

Eli Lilly and Company explored the 2-(azetidinyl)-pyridine scaffold and identified compound 24 as a potent starting point (see Fig. 7), exhibiting strong hKHK-C inhibitory activity in both biochemical and cellular assays. To further optimize the compound, the 1*H*-pyrazole moiety was extended with secondary amines, leading to compound 25 containing an azetidine substituent. The modification improved potency and solubility but was found to be toxic in rat hepatocytes (LC_20_ < 50 μM). A hydroxyl substituent was introduced to reduce the metabolic liability associated with the 2-methyl azetidine group, resulting in compound 26. Although this strategy reduced metabolic concerns but displayed high *in vivo* clearance. Subsequent optimization involved the replacement of the azetidine ring with a piperidine moiety in the pyrazole side chain. Although, compound 27 with this modification exhibited improved potency and metabolic stability but poor aqueous solubility.

**Fig. 7:**
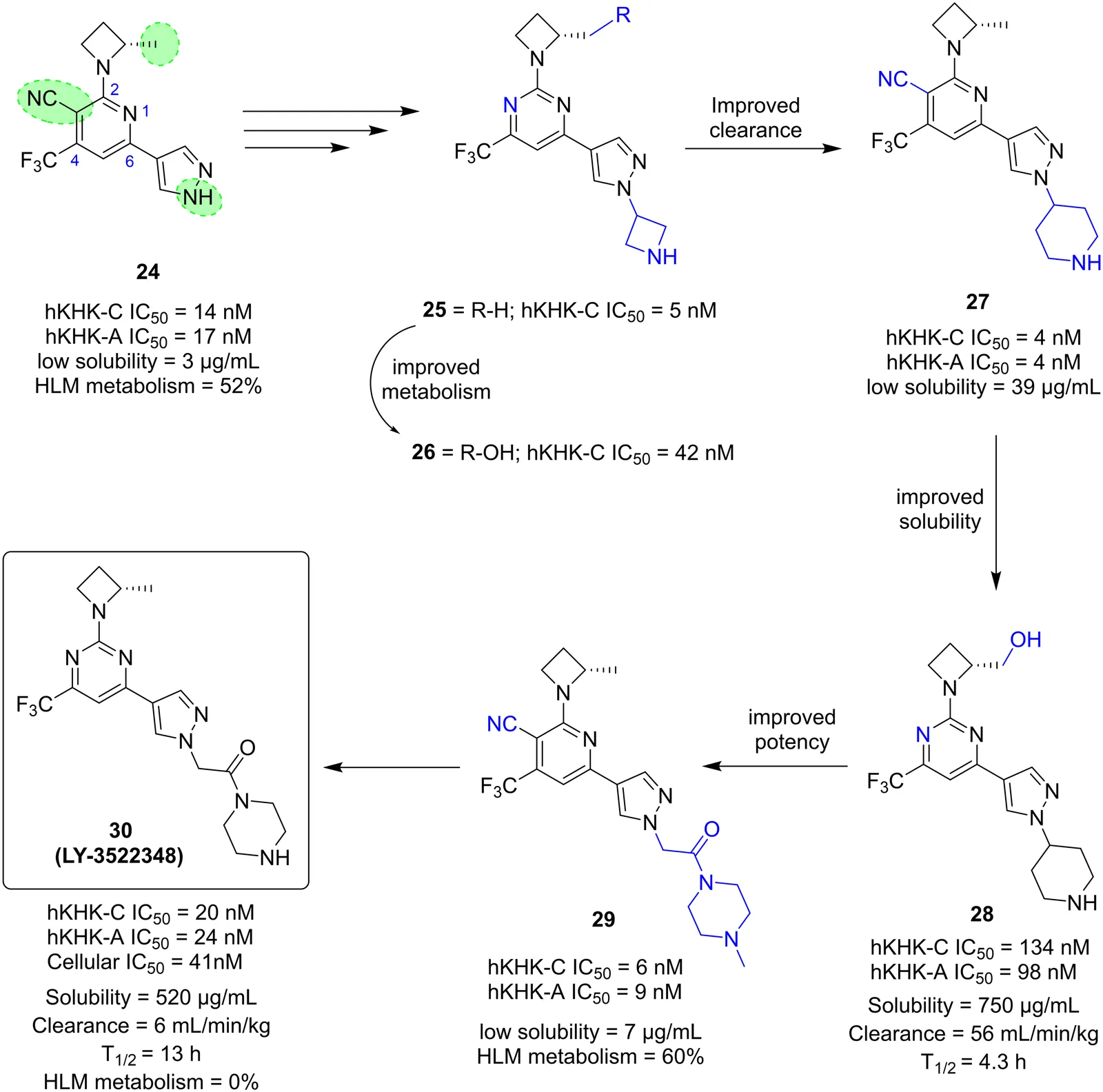
Development of the clinical candidate LY-3522348 by Eli Lilly and Company.

To further improve aqueous solubility, the nicotinonitrile core was replaced with a pyrimidine scaffold, and a hydroxylated azetidine substituent was introduced to reduce metabolic clearance. Compound 28 demonstrated improved potency, safety *in vitro*, and metabolic stability, and was therefore advanced to pharmacokinetic studies. Despite these improvements, compound 28 failed due to high clearance and short half-life. In further structural refinements, compounds 29 and 30 were obtained by inserting amide linkers between the pyrazole and polar secondary amines. Both compounds showed excellent biochemical potency; however, compound 29 lacked adequate metabolic stability and suboptimal *in vitro* safety. In contrast, compound 30 (LY-3522348) demonstrated better overall profile, including improved pharmacokinetic properties in both rat and dog studies.^32^ Same group disclosed a series of 2-(azetidinyl)pyridine-based KHK inhibitors in patent WO2020/051058 for the treatment of Metabolic Syndromes.^66^

Boehringer Ingelheim Pharma GmbH & Co. identified KHK inhibitors through virtual screening using the BICLAIM library.^67^ Starting from compound 31, approximately 10 000 compounds exhibiting similar 2D pharmacophoric features were selected. These were then refined through a 3D shape-similarity analysis, comparing them to the common pharmacophoric motifs of the compounds 4, 6, and 11.^68,69^ This led to the identification of hits 31 and 32 (see Fig. 8), which contained 2,6-diamino-3-cyano-4-(trifluoromethyl)pyridine core. Hybridization of compound 18 and 32 yielded 33 with nearly 10-fold greater potency (IC_50_ = 20.3 nM *vs.* 187 nM). Increasing the distance of the basic moiety from the core further improved the activity, resulting in 34 and 35 (IC_50_ = 5.5 and 4.2 nM, respectively). Despite its high potency, 35 exhibited poor off-target selectivity, inhibiting 22 out of 44 off-targets.

**Fig. 8:**
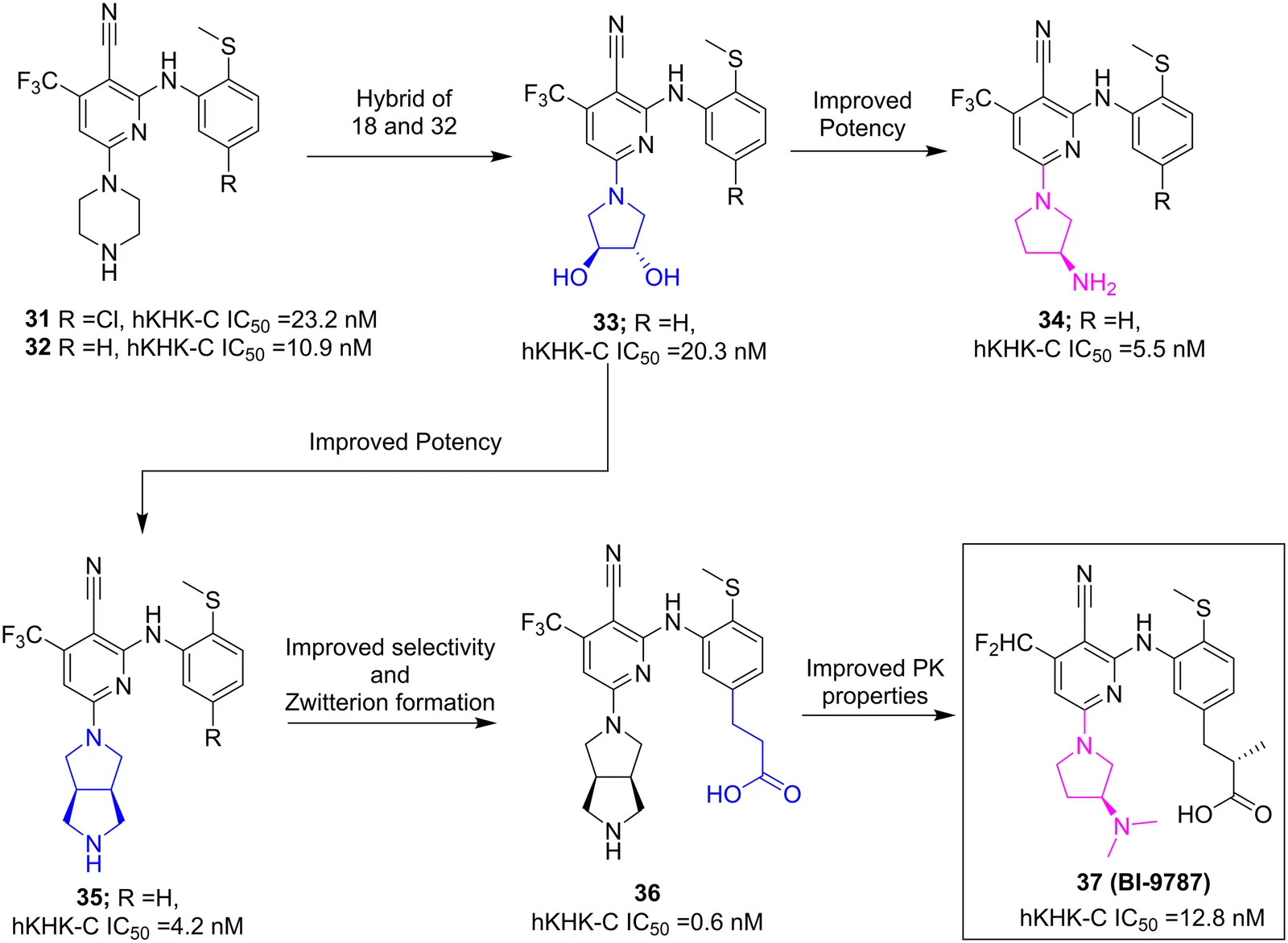
Development of GS-1291269 by Boehringer Ingelheim.

To improve selectivity and physicochemical properties, modifications were introduced at R of 35, reducing lipophilicity and allowing the incorporation of acidic functionalities without compromising potency. These changes generated zwitterionic analog 36, which exhibited improved selectivity, cellular activity, and microsomal stability. Nonetheless, its solubility and permeability were significantly compromised due to this modification. The diazabicyclooctane moiety was replaced with (*S*)-*N*,*N*-dimethylpyrrolidin-3-amine. Additionally, the introduction of α-methyl phenylpropionic acid showed increased lipophilicity and enhanced permeability. The optimized inhibitor 37 (BI-9787) displayed potent activity against hKHK-C (IC_50_ = 12.8 nM) and inhibited fructose-1-phosphate formation in HepG2 cells (IC_50_ = 123 nM).^33^ It also exhibited acceptable permeability and low aqueous solubility. In a selectivity panel of 44 unrelated targets, 37 showed more than 100-fold selectivity against all targets, except PDE4D2, for which the selectivity remained at least 30-fold. Same group disclosed a patent application (WO2024/121183) describing a series of 52 novel cyanopyridine-based ketohexokinase (KHK) inhibitors.^70^

PF-06835919 exhibited drug–drug interaction (DDI) liabilities in both preclinical and clinical studies, prompting further optimization by the Gilead Sciences team. To identify the minimal pharmacophoric elements required for pyrimidine-based KHK inhibitors, the authors used a truncated PF-06835919, which yielded compound 38 (see Fig. 9). This led to the discovery of an optimal core scaffold with compound 39 (IC_50_ = 2 nM). This was used as a starting point and was further explored for interaction at the KHK binding pocket, particularly with Asp27B. To achieve this, replacing the methyl group with a benzamide moiety at the 4-position did not compromise potency, yielding compound 40 (IC_50_ = 9 nM). Binding interactions showed that the amide end of benzamide formed an electrostatic interaction with Asp27B. Along with potency, compound 40 exhibited improved predicted clearance (predCL = 1.05 L h^−1^ kg^−1^) in human hepatocytes but surprisingly showed high clearance and low bioavailability in rat pharmacokinetic studies. The presence of a basic amine and low log *D* may have contributed to its poor PK properties. Further optimization led to compound 41 (IC_50_ = 6 nM), in which the fused cyclopentylpyrimidine ring was fluorinated and the azetidine moiety was hydroxylated. This provided improved predicted clearance (predCL = 0.26 L h^−1^ kg^−1^), *in vitro* metabolic stability, and reasonable bioavailability. However, despite showing favorable *in vitro* clearance, it exhibited poor clearance profiles across multiple species, indicating an *in vitro*–*in vivo* disconnect.

**Fig. 9:**
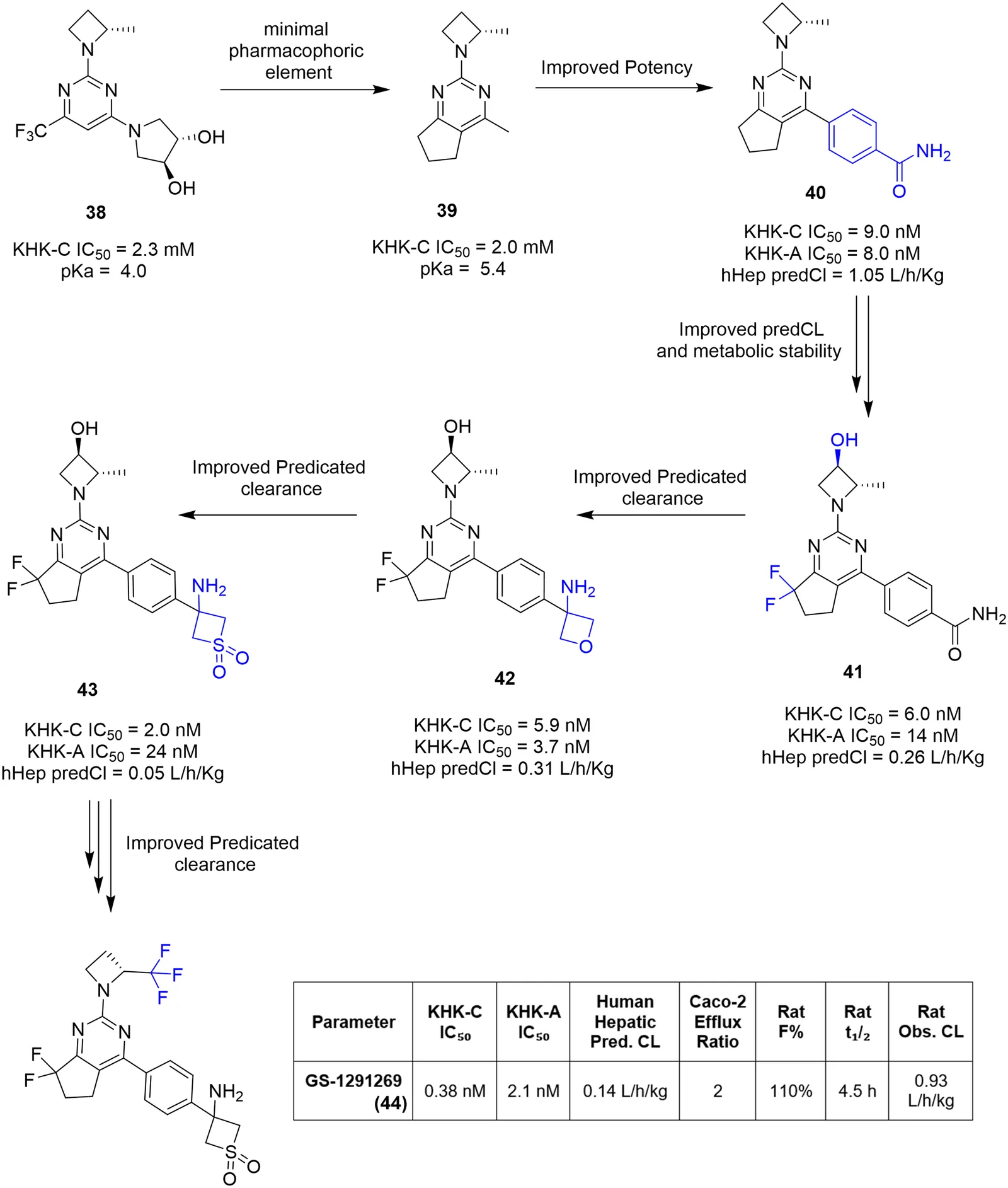
Development of the clinical candidate GS-1291269 by Gilead Sciences.

Various bioisosteric replacements of the benzamide functionality were explored to reduce the predicted clearance. Compound 42, containing an oxetan-3-amine as an amide bioisostere, showed good bioavailability and a reasonable half-life but continued to suffer from poor *in vivo* clearance (predCL = 0.31 L h^−1^ kg^−1^). Subsequently, compound 43, in which the oxetane oxygen was replaced with a sulfone group, also exhibited the same high *in vivo* clearance as the earlier analogs. In addition, compound 43 exhibited a very high Caco-2 efflux ratio of 3 : 1 indicating significant transporter-mediated efflux. SAR studies revealed that the azetidine alcohol group was a major contributor to efflux; thus, it was replaced with fluorinated motifs. This strategy led to GS-1291269 (44), which retained potency while improving the PK properties. GS-1291269 showed acceptable predicted clearance and a substantially reduced efflux ratio of 2, making it less likely to be subject to P-glycoprotein-mediated gastrointestinal clearance.^34^ Furthermore, it demonstrated excellent bioavailability across multiple species. No significant off-targets, transporter inhibition, or drug–drug interactions were observed. No publicly reported clinical trial results have been reported.

For a comparative summary of the major KHK inhibitors and their pharmacological profiles, see Table 1. From the KHK inhibitor optimization phase, it is apparent that focus should be on structural modifications that enhance the overall drug profile rather than solely increasing biochemical potency. This optimization process must achieve a balance between potency and various factors such as physicochemical properties, selectivity, permeability, metabolic stability, PK/PD characteristics, as well as toxicity and safety parameters. Enhancements in one attribute may adversely affect another, necessitating the simultaneous optimization of multiple parameters during lead optimization.^71,72^ Consequently, multiparameter optimization remains a pivotal strategy in medicinal chemistry for developing KHK inhibitors that are clinically viable.

Comparative summary of biochemical and cellular potency, pharmacokinetic properties, off-target selectivity, and isoform profiles of major KHK inhibitors (PF-06835919, LY-3522348, BI-9787, and GS-1291269)

**Table:** 

	PF-06835919 (ref. 31)	LY-3522348 (ref. 32)	BI-9787 (ref. 33)	GS-1291269 (ref. 34)
hKHK-C IC_50_ (nM)	28	20 ± 8	12.8	0.38
hKHK-A IC_50_ (nM)	170	24 ± 6	12	2.1
Cellular IC_50_ (nM)	—	41 ± 14	123^a^	—

**Table:** 

Species	Rat	Dog	Rat	Dog	Rat	Rat	Dog
*t*_½_ (h)^b^	7.71/5.7	5.57 ± 1.77/3.4	13/13	28/37	—	4.5	8.2
CL (mL min^−1^ kg^−1^)^c^	0.358	1.31 ± 0.55	6	3	15	15.5	16.33
Oral bioavailability, *F*% (dose, mg kg^−1^)	95 (5)	>100 (5)	83 (2)	87 (2)	68	100 (5)	198 (3)

a Cellular assay = F1P HepG2 IC_50_.

b Data given in the form of iv/po.

c IV dose – 1 mg kg^−1^.

### Clinical trial outcomes

To date, only two KHK inhibitors have progressed to clinical evaluation: PF-06835919, which advanced to phase II trials by Pfizer, and LY-3522348, which completed phase 1 trial under Eli Lilly (see Table 2). PF-06835919 has been reported to reduce hepatic triglyceride levels and improve insulin sensitivity, whereas LY-3522348 has demonstrated therapeutic potential in diabetes and non-alcoholic steatohepatitis (NASH).

**Table 2:** Summary of clinical trials of KHK inhibitors, including study phase and population, dose regimen, pharmacokinetic/pharmacodynamic findings, efficacy outcomes, and safety profile

Candidate	Trial	Phase; population	Dose-regimen	PK/PD findings	Efficacy finding	Safety
LY-3522348	NCT04559568	*N* = 65 healthy participants	SAD: 5–380 mg; MAD: 50–290 mg	*t*_½_ ∼24–34 h; dose-dependent inhibition of fructose metabolism – target engagement	Not an efficacy trial	Safe and well tolerated; no serious AEs or deaths
	Phase 1					
PF-06835919	NCT02974374	*N* = 16 healthy adults; randomized, double-blind, crossover SAD	SAD 10–600 mg	Dose-dependent exposure and KHK inhibition; ≥200 mg predicted ∼90% KHK inhibition	—	Single doses well tolerated; established human target engagement
	Phase 1					
	NCT03256526	*N* = 53 adults with NAFLD; randomized, placebo-controlled; 6 weeks	75 or 300 mg QD	Pharmacodynamic/metabolic effects consistent with KHK inhibition	300 mg reduced whole-liver fat by 18.73% relative to placebo (*p* = 0.04); 75 mg was not significant	No serious AEs
	Phase 2a					
	NCT03916406	*N* = 10 healthy participants; two-way crossover DDI study	300 mg QD × 9 days, then PF-06835919 + midazolam	Minimal change in midazolam AUC/*C*_max_; no evidence of clinically meaningful CYP3A4 induction	—	No serious AEs, deaths, or discontinuations
	Phase 1					
	NCT03969719	*N* = 164 participants with NAFLD + T2D; randomized, double-blind; 16 weeks	150 or 300 mg QD	Evidence of sustained KHK inhibition	WLF change: placebo – 5.26%; 150 mg – 17.05%; 300 mg – 19.13%; HbA1c improvement was not statistically significant	Safe and well tolerated; modest liver-fat/metabolic benefit
	Phase 2a					
	NCT04193436	*N* = 23 participants with normal to severe hepatic impairment	25 mg single dose	Exposure increased particularly with moderate/severe hepatic impairment	—	No serious AEs or deaths; characterized effect of hepatic impairment on PK
	Phase 1					
	NCT04427917	*N* = 8 healthy Japanese participants	300 mg QD × 7 days	AUC: 169.3 (day 1) – 230.9 μg h mL^−1^ (day 7); day-7 – *t*_½_ 14.62 h	—	Characterized multiple-dose PK in Japanese participants
	Phase 1					
	NCT03031119	*N* = 62	Results not publicly available			
	Phase 1	Multiple ascending-dose/DDI study				
	NCT05463575	*N* = 15	300 mg QD *vs.* placebo; 42 days	Designed to assess KHK inhibition and metabolic effects	Hepatic insulin sensitivity/metabolic endpoints	Results not publicly available
	Phase 2	NAFLD				

The initial phase 1 SAD/MAD study of LY-3522348 (NCT04559568) involved 65 healthy individuals to assess its safety, tolerability, and PK/PD profile.^73^ Dose-dependent exposure was observed at single doses of 5–380 mg and multiple once-daily doses of 50–290 mg for 14 days. LY-3522348 was observed well tolerated, and there were no reports of serious AEs or fatalities. PF-06835919 has undergone extensive clinical evaluation across phase 1 and phase 2 studies. The first-in-human phase 1 study (NCT02974374) evaluated single ascending oral doses of 10–600 mg in 16 healthy participants. Systemic exposure increased with increasing dose, with a terminal half-life of approximately 11–15 h across the evaluated dose range. Importantly, plasma fructose exposure following a fructose challenge increased dose-dependently, providing pharmacodynamic evidence of KHK inhibition. At dose of 200 mg and above, KHK inhibition was predicted to be approximately 90%. It was observed that single doses were generally well tolerated, with no severe adverse events (AEs) reported. These findings established human target engagement of PF-06835919 in patients with metabolic liver disease and supported its further evaluation. Additional phase 1 studies further characterized the clinical pharmacology of PF-06835919. The study on drug–drug interactions (NCT03916406; *N* = 10) demonstrated that administering PF-06835919 multiple times had negligible impact on midazolam exposure with no significant CYP3A4 induction.^74^ The pharmacokinetics of a single 25 mg dose were assessed in a hepatic impairment study (NCT04193436). This study included participants with mild, moderate, and severe liver impairment (*N* = 23) offering insights into alterations in both systemic and unbound drug exposure. A study involving multiple doses was conducted with healthy Japanese participants (NCT04427917) to examine the pharmacokinetics and tolerability of PF-06835919 administered at a daily dose of 300 mg. On day 1, the AUC was 169.3 μg h mL^−1^, which rose to 230.9 μg h mL^−1^ by day 7, and the terminal half-life recorded on day 7 was 14.62 hours.

A randomized, placebo-controlled, double-blind, phase 2a study involving 53 adults with NAFLD was conducted to evaluate the efficacy of PF-06835919 (NCT03256526).^64^ Participants received placebo or PF-06835919 at 75 or 300 mg once daily over a 6-week period. There was an 18.73% reduction in whole-liver fat with 300 mg compared to placebo (*p* = 0.04). Whereas the 75 mg dose did not produce a significant reduction. Improvements in inflammatory markers were also observed with no serious adverse events reported. The findings provided early clinical evidence that KHK inhibition could lead to a reduction in hepatic fat. A subsequent phase 2a study (NCT03969719) evaluated PF-06835919 in participants with NAFLD and type 2 diabetes mellitus.^75^ The study involved 164 participants who received PF-06835919 at a dose of 150 or 300 mg once daily for 16 weeks. It was observed that whole-liver fat decreased by 5.26% with placebo, 17.05% with 150 mg, and 19.13% with 300 mg of PF-06835919. Inhibition of KHK led to greater reductions in hepatic fat; however, there was no statistically significant effect on HbA1c. This suggests that KHK inhibition primarily affected hepatic fat rather than glycemic control. PF-06835919 was generally safe and well tolerated during the 16-week treatment period. Collectively, these phase 2a studies indicated that KHK inhibition effectively reduces hepatic fat. However, target engagement is not necessarily associated with comparable improvements for all metabolic endpoints. PF-06835919 was evaluated in a study involving multiple ascending doses (NCT03031119) as well as in a phase 2 trial targeting KHK inhibition in NAFLD (NCT05463575). Nonetheless, specific findings from these studies were not accessible to the public when the literature search was conducted.

## 7. Challenges in KHK inhibitor development

### Preclinical-to-clinical translational gap

Although KHK inhibition has shown strong effectiveness in preclinical models, the clinical effects have been less pronounced. This discrepancy between preclinical and clinical results of KHK inhibition could be due to several factors.^76^ Some of these challenges may partly explain why only a few KHK inhibitors have entered clinical trials, and none have received FDA approval.

First, variations in fructose metabolism across species may partly explain these differences. Although fructolytic pathway is conserved, rodents and humans differ in several aspects of fructose metabolism. This includes intestinal and hepatic fructose handling, tissue distribution of fructose transporters, and uric acid metabolism.^77^ Mice have substantial capacity to process fructose *via* first-pass intestinal metabolism. In contrast, first-pass intestinal and hepatic metabolisms are relatively different in humans.

Secondly, the variation in fructose exposure between human subjects and experimental models might affect how they respond to KHK inhibition. Preclinical models typically encounter controlled and frequently high fructose loads. In contrast, dietary fructose exposure in humans exhibits significant variability, influenced by overall dietary patterns. Additionally, it is not only confined to dietary intake, as fructose may also be synthesized endogenously from glucose through the polyol pathway.^78,79^ The relative contributions of dietary and endogenous fructose metabolism can vary significantly among patients.

Lastly, the heterogeneity and multifactorial nature of FDMDs may influence the variability in clinical responses to KHK inhibition.^31,80^ Patients with FDMDs exhibit significant differences in obesity, insulin resistance, type 2 diabetes, genetic predisposition, dietary habits, disease severity, and stage of progression. Beyond fructose metabolism, various interconnected mechanisms, such as insulin resistance, altered lipid metabolism, mitochondrial dysfunction, oxidative stress, *de novo* lipogenesis, and inflammation, play roles in the development and progression of FDMDs. Consequently, the contribution of fructose metabolism to disease pathology and the response to KHK inhibition may vary among patients. Therefore, all these differences should be considered when translating the metabolic benefits of KHK inhibition from animal studies to humans.

### Isoform- and tissue-specific selectivity

As KHK-C is primarily responsible for rapid fructose metabolism and associated metabolic abnormalities. The dual KHK-A/KHK-C inhibitor (LY-3522348) has demonstrated pharmacodynamic target engagement with acceptable human tolerability. However, isoform selectivity remains challenging because of the highly conserved catalytic sites of KHK-A and KHK-C. As discussed earlier, the alternatively spliced region alters the β-clasp/domain organization, through which isoform selectivity can be structurally achieved. This is supported by structural studies demonstrating that the two isoforms can be pharmacologically discriminated. PF-06835919 showed approximately 6-fold selectivity for KHK-C; in contrast, a structurally distinct inhibitor reported by Ebenhoch *et al.* showed approximately 50-fold selectivity for KHK-A.^54^ Genetic studies also suggest that selective KHK-C inhibition may be beneficial because KHK-A may contribute to fructose clearance. Besides its role in fructose metabolism, KHK-A has been reported to have other non-canonical functions. There is evidence that KHK-A can promote nucleotide synthesis and tumor growth and is also implicated in cardiac remodeling. These biological functions of KHK isoforms beyond fructose metabolism should also be considered while developing isoform-selective inhibitors.^81^ Alternatively, dual inhibition of KHK-A/KHK-C may be beneficial where KHK-A contributes to disease pathology. However, there is currently no strong clinical evidence establishing the therapeutic advantage of isoform selectivity.

Thus, it remains unclear whether selective KHK-C inhibition has a therapeutic advantage over dual KHK-A/KHK-C inhibition. Furthermore, preclinical studies indicate that the effect of fructose metabolism by KHK inhibition are tissue specific. Fructose-induced steatosis and metabolic dysfunction are particularly driven by hepatic KHK. On the other hand, intestinal KHK is involved in first-pass fructose clearance, whereas renal KHK is active in interorgan fructose metabolism.^81^ These findings suggest that KHK inhibitor development may benefit from tissue-specific targeting together with isoform selectivity.

## 8. Summary and future direction

In summary, the adoption of a Westernized lifestyle and the growing consumption of highly processed foods have led to increased fructose intake. Excessive and unregulated fructose metabolism contributes to several metabolic disorders, including NFALD/NASH, type 2 diabetes, chronic kidney disease, and other FDMDs. KHK is a key enzyme in fructose metabolism and has been validated as a promising therapeutic target. Additionally, the development of structurally distinct KHK inhibitors has progressed from hits to preclinical/clinical stages and has shown potential in treating FDMDs. Although the reported chemotypes differ considerably in structural complexity and show similar interaction profiles, KHK inhibitor lead optimization programs reveal several common challenges.

Several compounds demonstrating strong *in vitro* activity were found to have suboptimal pharmacokinetic profiles, suggesting that biochemical potency alone is insufficient for successful lead development. Consequently, future medicinal chemistry efforts should prioritize multiparameter optimization, which involves balancing potency with physicochemical and ADMET properties, permeability, transporter-mediated efflux, adequate target-tissue exposure, and PK/PD profiles. Given the challenges related to isoform and tissue selectivity, further research is necessary to ascertain whether selective KHK-C inhibition provides an advantage over dual KHK-A/KHK-C inhibition and whether tissue-specific targeting can enhance therapeutic outcomes. Additionally, the observed gap between preclinical and clinical translation underscores the need for improved translational models, suitable pharmacodynamic biomarkers, and the identification of patient populations with a higher reliance on fructose metabolism. Moreover, as FDMDs are multifactorial, KHK inhibition may offer greater benefits when used in combination therapies targeting complementary metabolic pathways. Moreover, expanding chemical diversity through AI/ML-assisted medicinal chemistry and drug design approaches may accelerate the discovery and development of next-generation KHK inhibitors for the treatment of FDMDs.

## Author contributions

SVGA: literature review, writing – original draft, visualization. TN and AJM: literature review and writing. TN: graphical abstract. BUH: conceptualization, supervision, writing, review, and editing of the original draft. All authors discussed the manuscript and reviewed and approved the final version.

## Conflicts of interest

The authors have no competing interests to declare.

## Data Availability

No primary research results or new data were generated as part of this review.

## References

[cit1] Baker P., Machado P., Santos T., Sievert K., Backholer K., Hadjikakou M., Russell C., Huse O., Bell C., Scrinis G., Worsley A., Friel S., Lawrence M. (2020). Ultra-Processed Foods and the Nutrition Transition: Global, Regional and National Trends, Food Systems Transformations and Political Economy Drivers. Obes. Rev..

[cit2] Monteiro C. A., Moubarac J. C., Cannon G., Ng S. W., Popkin B. (2013). Ultra-Processed Products Are Becoming Dominant in the Global Food System. Obes. Rev..

[cit3] Ng S. W., Ostrowski J. D., Li K. P. (2017). Trends in Added Sugars from Packaged Beverages Available and Purchased by US Households, 2007-2012. Am. J. Clin. Nutr..

[cit4] Duffy E. W., Hall M. G., Dillman Carpentier F. R., Musicus A. A., Meyer M. L., Rimm E., Smith Taillie L. (2021). Nutrition Claims on Fruit Drinks Are Inconsistent Indicators of Nutritional Profile: A Content Analysis of Fruit Drinks Purchased by Households With Young Children. J. Acad. Nutr. Diet..

[cit5] Ng S. W., Slining M. M., Popkin B. M. (2012). Use of Caloric and Noncaloric Sweeteners in US Consumer Packaged Foods, 2005-2009. J. Acad. Nutr. Diet..

[cit6] Louie J. C. Y., Moshtaghian H., Rangan A. M., Flood V. M., Gill T. P. (2016). Intake and Sources of Added Sugars among Australian Children and Adolescents. Eur. J. Nutr..

[cit7] Jung S., Bae H., Song W. S., Jang C. (2022). Dietary Fructose and Fructose-Induced Pathologies. Annu. Rev. Nutr..

[cit8] Popkin B. M., Hawkes C. (2016). Sweetening of the Global Diet, Particularly Beverages: Patterns, Trends, and Policy Responses. Lancet Diabetes Endocrinol..

[cit9] Bray G. A. (2013). Energy and Fructose From Beverages Sweetened With Sugar or High-Fructose Corn Syrup Pose a Health Risk for Some People. Adv. Nutr..

[cit10] High Fructose Corn Syrup Questions and Answers | FDA, https://www.fda.gov/food/food-additives-petitions/high-fructose-corn-syrup-questions-and-answers, (accessed 2026-07-11)

[cit11] Bray G. A., Nielsen S. J., Popkin B. M. (2004). Consumption of High-Fructose Corn Syrup in Beverages May Play a Role in the Epidemic of Obesity. Am. J. Clin. Nutr..

[cit12] Basciano H., Federico L., Adeli K. (2005). Fructose, Insulin Resistance, and Metabolic Dyslipidemia. Nutr. Metab..

[cit13] Ayoub-Charette S., Liu Q., Khan T. A., Au-Yeung F., Blanco Mejia S., De Souza R. J., Wolever T. M. S., Leiter L. A., Kendall C., Sievenpiper J. L. (2019). Important Food Sources of Fructose-Containing Sugars and Incident Gout: A Systematic Review and Meta-Analysis of Prospective Cohort Studies. BMJ Open.

[cit14] Hannou S. A., Haslam D. E., McKeown N. M., Herman M. A. (2018). Fructose Metabolism and Metabolic Disease. J. Clin. Invest..

[cit15] Özer N., Dalmızrak Ö., Elabaid S., Öğüş H. (2025). Fructose and Insulin: A Sweet Sabotage Hypothesis?. Endocrine.

[cit16] Tappy L., Le K. A. (2010). Metabolic Effects of Fructose and the Worldwide Increase in Obesity. Physiol. Rev..

[cit17] Jensen T., Abdelmalek M. F., Sullivan S., Nadeau K. J., Green M., Roncal C., Nakagawa T., Kuwabara M., Sato Y., Kang D. H., Tolan D. R., Sanchez-Lozada L. G., Rosen H. R., Lanaspa M. A., Diehl A. M., Johnson R. J. (2018). Fructose and Sugar: A Major Mediator of Nonalcoholic Fatty Liver Disease. J. Hepatol..

[cit18] Basaranoglu M., Basaranoglu G., Sabuncu T., Sentürk H. (2013). Fructose as a Key Player in the Development of Fatty Liver Disease. World J. Gastroenterol..

[cit19] Sharma A., An S. (2026). Phosphofructokinase in Glycolysis: Bridging Enzymology and Cell Biology. Exp. Cell Res..

[cit20] Curry D. L. (1989). Effects of Mannose and Fructose on the Synthesis and Secretion of Insulin. Pancreas.

[cit21] Shapiro A., Tümer N., Gao Y., Cheng K. Y., Scarpace P. J. (2011). Prevention and Reversal of Diet-Induced Leptin Resistance with a Sugar-Free Diet despite High Fat Content. J. Geophys. Res. Oceans.

[cit22] Romero F. A., Jones C. T., Xu Y., Fenaux M., Halcomb R. L. (2020). The Race to Bash NASH: Emerging Targets and Drug Development in a Complex Liver Disease. J. Med. Chem..

[cit23] Jamnik J., Rehman S., Blanco Mejia S., De Souza R. J., Khan T. A., Leiter L. A., Wolever T. M. S., Kendall C. W. C., Jenkins D. J. A., Sievenpiper J. L. (2016). Fructose Intake and Risk of Gout and Hyperuricemia: A Systematic Review and Meta-Analysis of Prospective Cohort Studies. BMJ Open.

[cit24] Zgaga L., Theodoratou E., Kyle J., Farrington S. M., Agakov F., Tenesa A., Walker M., McNeill G., Wright A. F., Rudan I., Dunlop M. G., Campbell H. (2012). The Association of Dietary Intake of Purine-Rich Vegetables, Sugar-Sweetened Beverages and Dairy with Plasma Urate, in a Cross-Sectional Study. PLoS One.

[cit25] Kanbay M., Jensen T., Solak Y., Le M., Roncal-Jimenez C., Rivard C., Lanaspa M. A., Nakagawa T., Johnson R. J. (2016). Uric Acid in Metabolic Syndrome: From an Innocent Bystander to a Central Player. Eur. J. Intern. Med..

[cit26] Johnson R. J., Segal M. S., Sautin Y., Nakagawa T., Feig D. I., Kang D. H., Gersch M. S., Benner S., Sánchez-Lozada L. G. (2007). Potential Role of Sugar (Fructose) in the Epidemic of Hypertension, Obesity and the Metabolic Syndrome, Diabetes, Kidney Disease, and Cardiovascular Disease. Am. J. Clin. Nutr..

[cit27] Ishimoto T., Lanaspa M. A., Le M. P. T., Garcia G. E., Diggle C. P., MacLean P. S., Jackman M. R., Asipu A., Roncal-Jimenez C. A., Kosugia T., Rivarda C. J., Maruyamad S., Rodriguez-Iturbe B., Sánchez-Lozada L. G., Bonthron D. T., Sauting Y. Y., Johnson R. J. (2012). Opposing Effects of Fructokinase C and A Isoforms on Fructose-Induced Metabolic Syndrome in Mice. Proc. Natl. Acad. Sci. U. S. A..

[cit28] Mirtschink P., Krishnan J., Grimm F., Sarre A., Hörl M., Kayikci M., Fankhauser N., Christinat Y., Cortijo C., Feehan O., Vukolic A., Sossalla S., Stehr S. N., Ule J., Zamboni N., Pedrazzini T., Krek W. (2015). HIF-Driven SF3B1 Induces KHK-C to Enforce Fructolysis and Heart Disease. Nature.

[cit29] Softic S., Gupta M. K., Wang G. X., Fujisaka S., O'Neill B. T., Rao T. N., Willoughby J., Harbison C., Fitzgerald K., Ilkayeva O., Newgard C. B., Cohen D. E., Kahn C. R. (2017). Divergent Effects of Glucose and Fructose on Hepatic Lipogenesis and Insulin Signaling. J. Clin. Invest..

[cit30] Ishimoto T., Lanaspa M. A., Rivard C. J., Roncal-Jimenez C. A., Orlicky D. J., Cicerchi C., Mcmahan R. H., Abdelmalek M. F., Rosen H. R., Jackman M. R., Maclean P. S., Diggle C. P., Asipu A., Inaba S., Kosugi T., Sato W., Maruyama S., Sánchez-Lozada L. G., Sautin Y. Y., Hill J. O., Bonthron D. T., Johnson R. J. (2013). High-Fat and High-Sucrose (Western) Diet Induces Steatohepatitis That Is Dependent on Fructokinase. Hepatology.

[cit31] Futatsugi K., Smith A. C., Tu M., Raymer B., Ahn K., Coffey S. B., Dowling M. S., Fernando D. P., Gutierrez J. A., Huard K., Jasti J., Kalgutkar A. S., Knafels J. D., Pandit J., Parris K. D., Perez S., Pfefferkorn J. A., Price D. A., Ryder T., Shavnya A., Stock I. A., Tsai A. S., Tesz G. J., Thuma B. A., Weng Y., Wisniewska H. M., Xing G., Zhou J., Magee T. V. (2020). Discovery of PF-06835919: A Potent Inhibitor of Ketohexokinase (KHK) for the Treatment of Metabolic Disorders Driven by the Overconsumption of Fructose. J. Med. Chem..

[cit32] Durham T. B., Hao J., Spinazze P., Stack D. R., Toth J. L., Massey S., Mbofana C. T., Johnston R. D., Lineswala J. P., Wrobleski A., Mínguez J. M., Perez C., Smith D. L., Lamar J., Leon R., Corkins C., Durbin J., Tung F., Guo S., Linder R. J., Yumibe N., Wang W., MacKrell J., Antonellis M., Mascaro B. (2023). Identification of LY3522348: A Highly Selective and Orally Efficacious Ketohexokinase Inhibitor. J. Med. Chem..

[cit33] Heine N., Weber A., Pautsch A., Gottschling D., Uphues I., Bauer M., Ebenhoch R., Magarkar A., Nosse B., Kley J. T. (2024). Discovery of BI-9787, a Potent Zwitterionic Ketohexokinase Inhibitor with Oral Bioavailability. Bioorg. Med. Chem. Lett..

[cit34] Kasun Z. A., Liang X., Ferrao R. D., Kaplan J. A., Clark C. T., Neubig M. E., Byun D. H., Badal S. S., Sroda N., Mistry T., Stanley N. H., Stevens K. L., Bachman J. L., Lo J. R., Loyer-Drew J., Velasquez M., Hao J., Mwangi J., Stafford B., Jansa P. (2026). The Discovery of GS-1291269: A Neutral Ketohexokinase (KHK) Inhibitor with an Unusual Thietane Amine Functional Group. J. Med. Chem..

[cit35] Mor I., Cheung E. C., Vousden K. H. (2011). Control of Glycolysis through Regulation of PFK1: Old Friends and Recent Additions. Cold Spring Harbor Symp. Quant. Biol..

[cit36] Cori G. T., Ochoa S., Slein M. W., Cori C. F. (1951). The Metabolism of Fructose in Liver. Isolation of Fructose-1-Phosphate and Inorganic Pyrophosphate. Biochim. Biophys. Acta.

[cit37] Lenzen S. (2014). A Fresh View of Glycolysis and Glucokinase Regulation: History and Current Status. J. Biol. Chem..

[cit38] Nelson D. L. and Cox M. M., PRINCIPLES OF BIOCHEMISTRY, Pearson Prentice Hall, 4th edn, 2006

[cit39] Schwarz J. M., Noworolski S. M., Wen M. J., Dyachenko A., Prior J. L., Weinberg M. E., Herraiz L. A., Tai V. W., Bergeron N., Bersot T. P., Rao M. N., Schambelan M., Mulligan K. (2015). Effect of a High-Fructose Weight-Maintaining Diet on Lipogenesis and Liver Fat. J. Clin. Endocrinol. Metab..

[cit40] Bode J. C., Zelder O., Rumpelt H. J., Wittkampy U. (1973). Depletion of Liver Adenosine Phosphates and Metabolic Effects of Intravenous Infusion of Fructose or Sorbitol in Man and in the Rat. Eur. J. Clin. Invest..

[cit41] Porte D., Baskin D. G., Schwartz M. W. (2002). Leptin and Insulin Action in the Central Nervous System. Nutr. Rev..

[cit42] Nogueiras R., Tschöp M. H., Zigman J. M. (2008). Central Nervous System Regulation of Energy Metabolism: Ghrelin versus Leptin. Ann. N. Y. Acad. Sci..

[cit43] Layden B. T., Durai V., Lowe Jr. W. L. (2010). G-Protein-Coupled Receptors, Pancreatic Islets, and Diabetes. Nat. Educ..

[cit44] Horvath T. L., Diano S., Sotonyi P., Heiman M., Tschöp M. (2001). Minireview: Ghrelin and the Regulation of Energy Balance—A Hypothalamic Perspective. Endocrinology.

[cit45] Skoracka K., Hryhorowicz S., Schulz P., Zawada A., Ratajczak-Pawłowska A. E., Rychter A. M., Słomski R., Dobrowolska A., Krela-Kaźmierczak I. (2025). The Role of Leptin and Ghrelin in the Regulation of Appetite in Obesity. Peptides.

[cit46] Farooqi I. S., Keogh J. M., Kamath S., Jones S., Gibson W. T., Trussell R., Jebb S. A., Lip G. Y. H., O'rahilly S. (2001). Partial Leptin Deficiency and Human Adiposity. Nature.

[cit47] Teff K. L., Elliott S. S., Tschöp M., Kieffer T. J., Rader D., Heiman M., Townsend R. R., Keim N. L., D'Alessio D., Havel P. J. (2004). Dietary Fructose Reduces Circulating Insulin and Leptin, Attenuates Postprandial Suppression of Ghrelin, and Increases Triglycerides in Women. J. Clin. Endocrinol. Metab..

[cit48] KHK - Ketohexokinase - Homo sapiens (Human) | UniProtKB, https://www.uniprot.org/uniprotkb/P50053/entry, (accessed 2026-10-03)

[cit49] Park J., Gupta R. S. (2008). Adenosine Kinase and Ribokinase - The RK Family of Proteins. Cell. Mol. Life Sci..

[cit50] Hayward B. E., Bonthron D. T. (1998). Structure and Alternative Splicing of the Ketohexokinase Gene. Eur. J. Biochem..

[cit51] Trinh C. H., Asipu A., Bonthron D. T., Phillips S. E. V. (2009). Structures of Alternatively Spliced Isoforms of Human Ketohexokinase. Acta Crystallogr., Sect. D:Biol. Crystallogr..

[cit52] Diggle C. P., Shires M., Leitch D., Brooke D., Carr I. M., Markham A. F., Hayward B. E., Asipu A., Bonthron D. T. (2009). Ketohexokinase: Expression and Localization of the Principal Fructose-Metabolizing Enzyme. J. Histochem. Cytochem..

[cit53] Sigrell J. A., Cameron A. D., Jones T. A., Mowbray S. L. (1998). Structure of Escherichia Coli Ribokinase in Complex with Ribose and Dinucleotide Determined to 1.8 Å Resolution: Insights into a New Family of Kinase Structures. Structure.

[cit54] Ebenhoch R., Bauer M., Romig H., Gottschling D., Kley J. T., Heine N., Weber A., Uphues I., Nar H., Pautsch A. (2023). Crystal Structures of Human and Mouse Ketohexokinase Provide a Structural Basis for Species- and Isoform-Selective Inhibitor Design. Acta Crystallogr., Sect. D:Struct. Biol..

[cit55] Ferreira J. C., Villanueva A. J., Fadl S., Al Adem K., Cinviz Z. N., Nedyalkova L., Cardoso T. H. S., Andrade M. E., Saksena N. K., Sensoy O., Rabeh W. M. (2024). Residues in the Fructose-Binding Pocket Are Required for Ketohexokinase-A Activity. J. Biol. Chem..

[cit56] Doke T., Ishimoto T., Hayasaki T., Ikeda S., Hasebe M., Hirayama A., Soga T., Kato N., Kosugi T., Tsuboi N., Lanaspa M. A., Johnson R. J., Kadomatsu K., Maruyama S. (2018). Lacking Ketohexokinase-A Exacerbates Renal Injury in Streptozotocin-Induced Diabetic Mice. Metabolism.

[cit57] Lanaspa M. A., Andres-Hernando A., Orlicky D. J., Cicerchi C., Jang C., Li N., Milagres T., Kuwabara M., Wempe M. F., Rabinowitz J. D., Johnson R. J., Tolan D. R. (2018). Ketohexokinase C Blockade Ameliorates Fructose-Induced Metabolic Dysfunction in Fructose-Sensitive Mice. J. Clin. Invest..

[cit58] Gibbs A. C., Abad M. C., Zhang X., Tounge B. A., Lewandowski F. A., Struble G. T., Sun W., Sui Z., Kuo L. C. (2010). Electron Density Guided Fragment-Based Lead Discovery of Ketohexokinase Inhibitors. J. Med. Chem..

[cit59] Zhang X., Song F., Kuo G. H., Xiang A., Gibbs A. C., Abad M. C., Sun W., Kuo L. C., Sui Z. (2011). Optimization of a Pyrazole Hit from FBDD into a Novel Series of Indazoles as Ketohexokinase Inhibitors. Bioorg. Med. Chem. Lett..

[cit60] Maryanoff B. E., O'Neill J. C., McComsey D. F., Yabut S. C., Luci D. K., Jordan A. D., Masucci J. A., Jones W. J., Abad M. C., Gibbs A. C., Petrounia I. (2011). Inhibitors of Ketohexokinase: Discovery of Pyrimidinopyrimidines with Specific Substitution
That Complements the ATP-Binding Site. ACS Med. Chem. Lett..

[cit61] Maryanoff B. E., O'Neill J. C., McComsey D. F., Yabut S. C., Luci D. K., Gibbs A. C., Connelly M. A. (2012). Pyrimidinopyrimidine Inhibitors of Ketohexokinase: Exploring the Ring C2 Group That Interacts with Asp-27B in the Ligand Binding Pocket. Bioorg. Med. Chem. Lett..

[cit62] Mayer M., Meyer B. (1999). Characterization of Ligand Binding by Saturation Transfer Difference NMR Spectroscopy. Angew. Chem., Int. Ed..

[cit63] Huard K., Ahn K., Amor P., Beebe D. A., Borzilleri K. A., Chrunyk B. A., Coffey S. B., Cong Y., Conn E. L., Culp J. S., Dowling M. S., Gorgoglione M. F., Gutierrez J. A., Knafels J. D., Lachapelle E. A., Pandit J., Parris K. D., Perez S., Pfefferkorn J. A., Price D. A., Raymer B., Ross T. T., Shavnya A., Smith A. C., Subashi T. A., Tesz G. J., Thuma B. A., Tu M., Weaver J. D., Weng Y., Withka J. M., Xing G., Magee T. V. (2017). Discovery of Fragment-Derived Small Molecules for in Vivo Inhibition of Ketohexokinase (KHK). J. Med. Chem..

[cit64] Kazierad D. J., Chidsey K., Somayaji V. R., Bergman A. J., Birnbaum M. J., Calle R. A. (2021). Inhibition of Ketohexokinase in Adults with NAFLD Reduces Liver Fat and Inflammatory Markers: A Randomized Phase 2 Trial. Med..

[cit65] Zhu G., Li J., Lin X., Zhang Z., Hu T., Huo S., Li Y. (2023). Discovery of a Novel Ketohexokinase Inhibitor with Improved Drug Distribution in Target Tissue for the Treatment of Fructose Metabolic Disease. J. Med. Chem..

[cit66] Durham T. B. , 2,6-Diamino Pyridine Compounds, WO2020/051058A1, 2020

[cit67] Lessel U., Wellenzohn B., Lilienthal M., Claussen H. (2009). Searching Fragment Spaces with Feature Trees. J. Chem. Inf. Model..

[cit68] Hawkins P. C. D., Skillman A. G., Nicholls A. (2007). Comparison of Shape-Matching and Docking as Virtual Screening Tools. J. Med. Chem..

[cit69] Hawkins P. C. D., Skillman A. G., Warren G. L., Ellingson B. A., Stahl M. T. (2010). Conformer Generation with OMEGA: Algorithm and Validation Using High Quality Structures from the Protein Databank and Cambridge Structural Database. J. Chem. Inf. Model..

[cit70] Joerg K. , Dirk G., Niklas H., Bernd N., Alexander P. and Alexander W., New Cyanopyridine KHK Inhibitor Compounds, WO2024/121183A1, 2024

[cit71] Cummins D. J., Bell M. A. (2016). Integrating Everything: The Molecule Selection Toolkit, a System for Compound Prioritization in Drug Discovery. J. Med. Chem..

[cit72] Hirlekar B. U., Nuthi A., Singh K. D., Murty U. S., Dixit V. A. (2023). An Overview of Compound Properties, Multiparameter Optimization, and Computational Drug Design Methods for PARP-1 Inhibitor Drugs. Eur. J. Med. Chem..

[cit73] Fukuda T., Thompson B. R., Brouwers B., Qian H. R., Wang W., Morse B. L., LaBell E. S., Durham T. B., Konig M., Haupt A., Benson C. T., MacKrell J. (2025). LY3522348, A New Ketohexokinase Inhibitor: A First-in-Human Study in Healthy Adults. Diabetes Ther..

[cit74] Qiu R., Fonseca K., Bergman A., Lin J., Tess D., Newman L., Fahmy A., Useckaite Z., Rowland A., Vourvahis M., Rodrigues D. (2024). Study of the Ketohexokinase Inhibitor PF-06835919 as a Clinical Cytochrome P450 3A Inducer: Integrated Use of Oral Midazolam and Liquid Biopsy. Clin. Transl. Sci..

[cit75] Saxena A. R., Lyle S. A., Khavandi K., Qiu R., Whitlock M., Esler W. P., Kim A. M. (2023). A Phase 2a, Randomized, Double-Blind, Placebo-Controlled, Three-Arm, Parallel-Group Study to Assess the Efficacy, Safety, Tolerability and Pharmacodynamics of PF-06835919 in Patients with Non-Alcoholic Fatty Liver Disease and Type 2 Diabetes. Diabetes, Obes. Metab..

[cit76] Herman M. A., Birnbaum M. J. (2021). Molecular Aspects of Fructose Metabolism and Metabolic Disease. Cell Metab..

[cit77] Jang C., Hui S., Lu W., Cowan A. J., Morscher R. J., Lee G., Liu W., Tesz G. J., Birnbaum M. J., Rabinowitz J. D. (2018). The Small Intestine Converts Dietary Fructose into Glucose and Organic Acids. Cell Metab..

[cit78] Hannou S. A., Haslam D. E., McKeown N. M., Herman M. A. (2018). Fructose Metabolism and Metabolic Disease. J. Clin. Invest..

[cit79] Lanaspa M. A., Ishimoto T., Li N., Cicerchi C., Orlicky D. J., Ruzycki P., Rivard C., Inaba S., Roncal-Jimenez C. A., Bales E. S., Diggle C. P., Asipu A., Petrash J. M., Kosugi T., Maruyama S., Sanchez-Lozada L. G., McManaman J. L., Bonthron D. T., Sautin Y. Y., Johnson R. J. (2013). Endogenous Fructose Production and Metabolism in the Liver Contributes to the Development of Metabolic Syndrome. Nat. Commun..

[cit80] Hou R., Panda C., Voruganti V. S. (2019). Heterogeneity in Metabolic Responses to Dietary Fructose. Front. Genet..

[cit81] Zhang H., Zhu Y., Xu C. (2026). Ketohexokinase: A Central Mediator of Fructose-Associated Pathogenesis and Promising Therapeutic Target. Pharmacol. Res..

